# Medium-chain fatty acids enhance expression and histone acetylation of genes related to lipid metabolism in insulin-resistant adipocytes

**DOI:** 10.1016/j.bbrep.2021.101196

**Published:** 2022-01-05

**Authors:** Musashi Kawamura, Naoki Goda, Natsuyo Hariya, Mayu Kimura, Shiori Ishiyama, Takeo Kubota, Kazuki Mochizuki

**Affiliations:** a Graduate School of Life and Environmental Sciences, University of Yamanashi, 4-4-37 Takeda, Kofu, Yamanashi, 400-8510, Japan; b Faculty of Life and Environmental Sciences, University of Yamanashi, 4-4-37 Takeda, Kofu, Yamanashi, 400-8510, Japan; c Department of Nutrition, Faculty of Health and Nutrition, Yamanashi Gakuin University, 2-4-5, Sakaori, Kofu, Yamanashi, 400-8575, Japan; d Department of Integrated Applied Life Science, Integrated Graduate School of Medicine, Engineering, and Agricultural Sciences, University of Yamanashi, 4-4-37 Takeda, Kofu, Yamanashi, 400-8510, Japan; e Department of Child Studies, Faculty of Child Studies, Seitoku University, 550, Iwase, Matsudo, Chiba, 271-8555, Japan

**Keywords:** Histone acetylation, Medium-chain fatty acid, Short-chain fatty acid, Insulin resistance, Adipocyte

## Abstract

**Background:**

The expressions of genes related to lipid metabolism are decreased in adipocytes with insulin resistance. In this study, we examined the effects of fatty acids on the reduced expressions and histone acetylation of lipid metabolism-related genes in 3T3-L1 adipocytes treated with insulin resistance induced by tumor necrosis factor (TNF)-α.

**Methods:**

Short-, medium-, and long-chain fatty acid were co-administered with TNF-α in 3T3-L1 adipocytes. Then, mRNA expressions and histone acetylation of genes involved in lipid metabolism were determined using mRNA microarrays, qRT-PCR, and chromatin immunoprecipitation assays.

**Results:**

We found in microarray and subsequent qRT-PCR analyses that the expression levels of several lipid metabolism-related genes, including *Gpd1*, *Cidec*, and *Cyp4b1*, were reduced by TNF-α treatment and restored by co-treatment with a short-chain fatty acid (C4: butyric acid) and medium-chain fatty acids (C8: caprylic acid and C10: capric acid). The pathway analysis of the microarray showed that capric acid enhanced mRNA levels of genes in the PPAR signaling pathway and adipogenesis genes in the TNF-α-treated adipocytes. Histone acetylation around *Cidec* and *Gpd1* genes were also reduced by TNF-α treatment and recovered by co-administration with short- and medium-chain fatty acids.

**General significance:**

Medium- and short-chain fatty acids induce the expressions of *Cidec* and *Gpd1*, which are lipid metabolism-related genes in insulin-resistant adipocytes, by promoting histone acetylation around these genes.

## Highlights

• Expressions of lipid metabolism genes are reduced in insulin-resistant adipocytes.
• Short- and medium-chain fatty acids inhibit lipid metabolism gene downregulation.
• Capric acid enhances expressions of PPAR signaling and adipogenesis genes.
• This mechanism involves recovery of histone acetylation in lipid metabolism genes.

## 1 Introduction

Insulin resistance is known to be a strong risk factor for the development of lifestyle-related diseases, such as type 2 diabetes mellitus. These are caused by several lifestyle factors, including dietary habits. Recent studies have explored the development of insulin resistance using adipocyte culture cell lines, such as 3T3-L1 adipocytes. It has been reported that insulin resistance inducers, including tumor necrosis factor (TNF)-α, reduced the activities and/or expressions of lipoprotein lipase (*Lpl*), an enzyme associated with triglyceride uptake in adipocytes [1,2]; glycerol-3-phosphate dehydrogenase 1 (*Gpd1*) and diacylglycerol transferase 1 (*Dgat1*), enzymes involved in triglyceride synthesis [3,4]; *Cidec*, an enzyme involved in lipid droplet production [5]; and adiponectin (*Adipoq*), an insulin sensitivity hormone [6]. A recent study demonstrated that insulin resistance induced by TNF-α in 3T3-L1 adipocytes was restored by rosiglitazone, an agonist of the peroxisome proliferator-activated receptor (PPAR) γ2 (PPARG2), an important nuclear receptor for adipocyte differentiation [7]. Therefore, the development of insulin resistance in adipocytes may be attributed to reduced PPARG2 activity.

Insulin resistance may be regulated by epigenetic modifications, such as histone modifications and DNA methylation. Our recent studies demonstrated that the downregulation of *Adipoq* and *Lpl* expressions caused by TNF-α administration in 3T3-L1 adipocytes was associated with reduced histone acetylation [6,8]. This results in the conversion of heterochromatin to euchromatin and induction of transcriptional responses through the recruitment of transcriptional complexes onto target genes [[9], [10], [11]]. These results indicate that development of insulin resistance in adipocytes may be regulated by epigenetic histone acetylation.

Recent studies have shown that histone acetylation is enhanced by inhibitors of histone deacetylases (HDACs), which are enzymes that eliminate acetylation on histones [12]. Earlier studies demonstrated that HDAC activity was decreased in differentiating adipocytes [13,14]. On the other hand, short-chain fatty acids, including butyric acid, are known to be HDAC inhibitors (HDACis) that enhance adipocyte differentiation and expressions of associated transcription factors, such as PPARG and CCAAT/enhancer binding protein α (C/EBPα) [15]. Thus, short- and medium-chain fatty acids are dietary factors that may effectively induce histone acetylation.

Short- and medium-chain fatty acids have carbon numbers of <8 and 8–12, respectively. In foods, the major short-chain fatty acid is butyric acid (C4), and the major medium-chain fatty acids are caprylic acid (C8) and capric acid (C10). Intake of medium-chain fatty acids was reported to enhance production of pyruvic acid, ketone bodies, and β-hydroxybutyric acid, which is an HDACi [16]. A study demonstrated that caprylic acid enhanced histone H3K9 acetylation in the promoter regions of beta-defensin 1 (*Pbd1*) and *Pbd2* genes in macrophage-like cells [17]. Medium-chain fatty acids also provide more acetyl-CoA and β-hydroxybutyric acid than short-chain fatty acids due to their higher carbon numbers. On the other hand, the major source of short-chain fatty acids in foods is dietary fibers metabolized in the large intestine. However, these short-chain fatty acids are mostly used as energy sources in the large intestine. No studies have examined whether medium-chain fatty acids induced histone acetylation around metabolic genes and ameliorated the reduced expressions of metabolic genes in insulin-resistant adipocytes.

In this study, we investigated whether a short-chain fatty acid (butyric acid) and medium-chain fatty acids (caprylic acid and capric acid) restored the reduced expressions of lipid metabolic genes induced by treatment with TNF-α in 3T3-L1 adipocytes. Furthermore, we identified whether these ameliorations were associated with increased acetylation of histones H3 and H4 around these lipid metabolic genes.

## 2 Materials and methods

### 2.1 Cell culture

For cell culture, 3T3-L1 preadipocyte cells were obtained from American Type Culture Collection (Manassas, VA). The cells were cultured at 37 °C in a humidified atmosphere with 5% CO_2_ in Dulbecco's modified Eagle's medium (DMEM) supplemented with high glucose (#D6429-500 ML, Sigma-Aldrich, St. Louis, MO) containing 10% calf bovine serum (MP Biomedicals, Santa Ana, CA), 2 mM glutamine, 20 mM Hepes (pH 7.4), non-essential amino acids solution (Sigma Aldrich), and antibiotic-antifungal agent solution (Nacalai Tesque, Tokyo, Japan). Adipogenic induction was performed by replacing the media with differentiation media, comprising DMEM supplemented with 10% FBS, 0.5 mM 3-isobutyl-1-methylxanthine (Sigma-Aldrich), 2 μM dexamethasone (Wako Pure Chemical Industries Ltd., Osaka, Japan), and 1.7 μM bovine insulin (derived from bovine pancreas; Sigma-Aldrich). After 96 h of stimulation, the cells were cultured in DMEM with 10% FBS. At 6 d post-adipogenic stimulation, co-treatment of the cells with fatty acids and TNF-α 3T3-L1 cells was performed in DMEM containing 10% FBS with various fatty acids (butyric acid, hexanoic acid, and palmitic acid [Wako Pure Chemical Industries]; caprylic acid, capric acid, and lauric acid [Nacalai Tesque]) and 5 ng/mL TNF-α (Peprotech, Rocky Hill, NJ) for 48 h. To achieve the same concentrations of dimethyl sulfoxide (DMSO) or BSA as the treatment groups, fatty acids dissolved in DMSO (0, 10, 20, 50, 200, or 1000 mM) were added at ratios of 1/1000 volume. Final concentrations in the media were then adjusted to 0, 10, 20, 50, 200, or 1000 μM with 0.1% DMSO. Then, 5 μg/mL TNF-α in 0.1% BSA were added at ratios of 1/1000 vol to final concentrations of 5 ng/mL TNF-α and 0.0001% BSA. Before addition of the TNF-α media, the media containing each fatty acid were sonicated for 1 min (range 1, MODEL Q55, QSONICA, Newtown, CT) and left to stand for at least 20 min to diffuse and dissolve each fatty acid.

### 2.2 qRT-PCR

Total RNA extraction and qRT-PCR were performed as previously described [15]. The cycle threshold (CT) values for the genes detected by qRT-PCR were converted to signal intensities using the delta-delta method [18]. The target mRNA levels were normalized with the corresponding transcription factor IIB (*Tf2b*) levels since variations in Ct values of *T2fb* in qRT-PCR are the lowest among several housekeeping genes, including *Tf2b*, TATA-Box Binding Protein (*Tbp*), eukaryotic initiation factor-4A (*Elf4a2*), cytochrome C1 (*Cyc 1*), hypoxanthine phosphoribosyltransferase (*Hprt*), actin beta (*Actb*), and glyceraldehyde-3-phosphate dehydrogenase (*Gapdh*). The formula used was: 2^(CT^ ^*T2Ib*^ ^– CT each gene)^. The sequences of the PCR primer pairs are shown in Supplemental Table S1.

### 2.3 Microarray analysis

Total RNA was extracted from four groups: cells without TNF-α or fatty acid treatment (BSA-Cont); cells treated with TNF-α only (T-Cont); cells treated with TNF-α and 1000 μM butyric acid (T-C4); and cells treated with TNF-α and 1000 μM capric acid (T-C10). Aliquots containing 100 ng of total RNA were individually converted to cDNA, fractionated, and labeled with a Gene ChIP® Poly-A RNA control kit, WT amplification kit, and Gene ChIP® WT terminal labeling kit (Affymetrix, Santa Clara, CA), according to the manufacturer's instructions. Hybridization, washing, and staining were performed using Affymetrix® MoGene2.1 ST array strips and a GeneAtlas® hybridization wash and Stain Kit for WT Assay Strips (Affymetrix), according to the manufacturer's protocols. After washing, the MoGene2.1 Array Strips were analyzed using a GeneAtlas imaging station (Affymetrix). Data analysis was performed using Expression Console (Affymetrix), Transcriptome Analysis Console (Affymetrix), and Excel (Microsoft) software. Pathway analyses were performed using WikiPathways (https://www.wikipathways.org/index.php/WikiPathways) on Transcriptome Analysis Console (Affymetrix). The inclusion criteria for the pathway analyses included genes with ≥1.5-fold upregulation and ≤−1.5-fold downregulation among genes with *p*-values<0.2 based on ANOVA. The genes detected in the pathway were confirmed by subsequent qRT-PCR.

### 2.4 MTT assay

MTT assay was performed as previously described [19]. Briefly, 3T3-L1 cells were cultured in DMEM containing 10% FBS with various fatty acids (butyric acid, hexanoic acid, and palmitic acid) at final concentrations of 0, 10, 20, 50, 200, or 1000 μM for 45 h. Adipocytes stimulated for 96 h were then continuously cultured in the same media with 0.1 mg/mL 1-(4,5-dimethylthiazol-2-yl)-3,5-diphenylformazan (MTT) for 3 h. The culture media were removed, and the cells were incubated in 0.1 mL buffer (38 mM HCl, 10% sodium dodecyl sulfate) overnight under a preventing light. MTT activity was then obtained by measuring absorbance at a wavelength of 440 nm.

### 2.5 Chromatin immunoprecipitation (ChIP) assay

Cell fixation and ChIP assays were performed as previously described [6]. The following specific antibodies were used: anti-acetyl-histone H3 at lysine 9 and 14 (Millipore, Billerica, MA), anti-acetyl-histone H4 at lysine 5, 8, 12 and 16 (Millipore), *anti*-PPARG (Cell Signaling Technology, Danvers, CA), and control rabbit IgG (Sigma Aldrich, Louis, MO). The CT values of the ChIP and input signals detected using qPCR were converted into signal intensities using the delta-delta method. Differences of 1 CT values were considered two-fold differences between samples [18]. All ChIP signals were normalized with the corresponding input signals. The following formula was used: 2^(CT input – CT acetylated histone or PPARG)^. The sequences of the PCR primer pairs are shown in Supplementary Table S2.

### 2.6 Statistical analysis

The results are expressed as mean ± standard error of mean (SEM). Significant differences were determined using Dunnett's test after one-way analysis of variance (ANOVA) for three or more groups or Student's *t*-test for two groups. *p-*values<0.05 were considered statistically significant.

## 3 Results

### 3.1 Effect of fatty acids on the expressions of lipid metabolism-related genes in TNF-α-treated 3T3-L1 adipocytes

First, we identified the optimal concentrations of fatty acids for inducing the expressions of lipid metabolism-related genes. The mRNA levels of *Fabp4*, a known adipocyte differentiation marker, were higher in the cells administered fatty acids (1000 μM) than in the cells administered DMSO (1000 μM). The mRNA levels of *Dgat2*, a gene related to triglyceride synthesis, were higher in the cells administered butyric acid (C4), hexanoic acid (C6), caprylic acid (C8), capric acid (C10), and lauric acid (C12) than in cells administered DMSO (1000 μM). This increase in *Dgat2* gene expression was observed to be dose-dependent (Supplementary Fig. S1). Through an MTT assay, we observed that cell viabilities were reduced by treatment with capric acid at 200 and 1000 μM. However, treatment with the other fatty acids did not significantly reduce cell viabilities (Supplementary Fig. S2).

Because short- and medium-chain fatty acids enhanced mRNA expression of *Fabp4* and *Dgat2* in 3T3-L1 adipocytes in a dose dependent manner, we evaluated gene expressions in adipocytes co-treated with the fatty acids at 1000 μM and TNF-α using microarray analysis. There were 81 and 41 genes with ≥0.8-fold and ≤-0.8-fold increases, respectively, in base 2 logarithm (1.74 in natural number) in the T-Cont compared with BSA-Cont. The total number of genes with altered expressions was 122. Among the 41 genes with ≤-0.8-fold increases, 5 and 6 genes in T-C4 and T-10, respectively, had significantly higher expressions than T-Cont. Among the 81 genes with ≥0.8-fold increases, 3 and 5 genes in T-C4 and T-C10, respectively, had significantly lower expression. We found genes related to metabolism, which were downregulated by TNF-α and upregulated by C4 (*Cidec*, *Gpd1*, and *Cyp4b1*) or C10 (*Cidec* and *Cyp4b1*), in the microarray analysis (Table 1).

**Table 1:** Expression changes detected by microarray analysis in cells co-treated with TNF-α and butyric acid (C4) or capric acid (C10).

C4	Function	Description	Gene	T-Cont vs. BSA-Cont		T-C4 vs.		Unigene ID
						T-Cont		
				Log2 ratio	*P*	Log2 ratio	*P*	
Down-regulation by TNF-α								
	Metabolism	Glycerol-3-phosphate dehydrogenase 1 (soluble)	*Gpd1*	−1.3	0.026	1.77	0.006	Mm.252,391
		Cytochrome P450, family 4, subfamily b, polypeptide 1	*Cyp4b1*	−1.5	0.004	1.57	0.005	Mm.1840
		Cell death-inducing DFFA-like effector c	*Cidec*	−1.51	0.035	2.06	0.03	Mm.10,026
	Immune response	Family with sequence similarity 213, member A	*Fam213a*	−0.83	0.004	1.74	0.035	Mm.27,227
		CD248 antigen, endosialin	*Cd248*	−1.74	0.032	1.66	0.039	Mm.29,597
Up-regulation by TNF-α								
	Immune response	Lymphocyte antigen 6 complex, locus E	*Ly6e*	0.92	0.006	−0.62	0.022	Mm.788
		Chemokine (C-X-C motif) ligand 11	*Cxcl11*	2.22	0.011	−1.58	0.011	Mm.131,723
	Transporter	Solute carrier family 25 (mitochondrial carrier), member 18	*Slc25a18*	1.86	0.014	−1.37	0.044	Mm.425,340
C10	Function	Description	Gene	T-Cont vs. BSA-Cont		T-C10 vs. T-Cont		Unigene ID
				Log2 ratio	*P*	Log2 ratio	*P*	
Down-regulation by TNF-α								
	Metabolism	Cytochrome P450, family 4, subfamily b, polypeptide 1	*Cyp4b1*	−1.5	0.004	2.51	0.014	Mm.1840
		Cell death-inducing DFFA-like effector c	*Cidec*	−1.51	0.035	6.68	0.041	Mm.10,026
	Immune response	Family with sequence similarity 213, member A	*Fam213a*	−0.83	0.004	4.53	0.031	Mm.27,227
	Signal transduction	Paternally expressed 10	*Peg 10*	−1.41	0.022	2.89	0.041	Mm.320,575
		Melanoma cell adhesion molecule	*Mcam*	−1.4	0.03	1.54	0.028	Mm.275,003
	Unknown	Predicted gene, 24,843	*Gm24843*	−1.23	0.04	1.91	0.02	
Up-regulation by TNF-α								
	Signal transduction	Mutated in colorectal cancers	*Mcc*	1	0.013	−0.87	0.024	Mm.312,511
		Bone marrow stromal cell antigen 1	*Bst 1*	1.08	0.005	−0.52	0.013	Mm.246,332
	Transcription/	Ankyrin repeat domain 1 (cardiac muscle)	*Ankrd1*	1.39	0.023	−1.79	0.022	Mm.10,279
	chromatin							

**Fig. 1:**
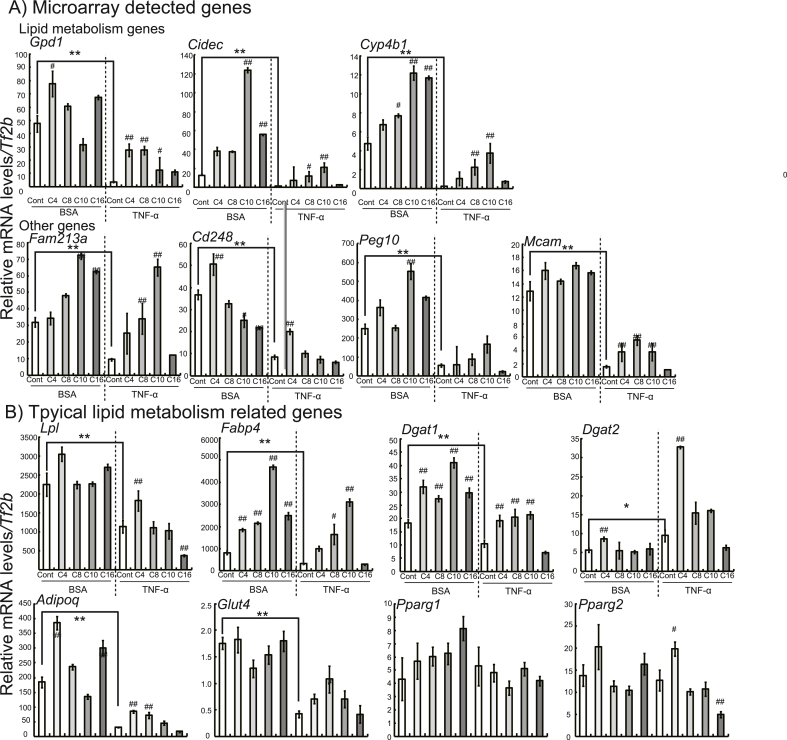
Effects of treatment with fatty acids (1000 μM) on the expressions of genes related to lipid metabolism in 3T3-L1 adipocytes with and without TNF-α administration. After reaching 80% confluence, 3T3-L1 cells were treated with adipocyte differentiation media for 96 h (regarded as day 0) and subsequently cultured in 10% FBS-containing DMEM for 6 d. The cells were then incubated with or without TNF-α (BSA only) and individual fatty acids (butyric acid [C4], caprylic acid [C8], capric acid [C10], or palmitic acid [C16]) for 48 h qRT-PCR was performed, with target mRNA levels normalized using *Tf2b* mRNA levels. The data are represented as the means ± SEM for the 6 plates. Statistical analyses for differences between two groups (BSA-Cont and T-Cont cells) were performed using Student's *t*-test (*P < 0.05, **P < 0.01). Statistical analyses for differences among three or more groups treated with fatty acids were carried out using Dunnett's test based on ANOVA (^#^*P* < 0.05, ^##^*P* < 0.01).

We then performed qRT-PCR on the genes detected in the microarray analysis, including *Cidec*, *Gpd1,* and *Cyp4b1* and other gene candidates. We found that all genes in candidates in microarray analysis had lower expression levels in TNF-α-treated cells than in BSA-treated cells, and treatment with butyric acid (*Gpd1*, *Cd248*, and *Mcam*), caprylic acid (*Gpd1*, *Cidec*, *Cyp4b1*, *Fam213a*, and *Mcam*), and capric acid (*Gpd1*, *Cidec*, *Cyp4b1*, *Fam213a*, *Cd248*, and *Mcam*), but not palmitic acid induced the expression of these genes in TNF-α-treated cells (Fig. 1A). Regarding typical lipid metabolism related genes, expression of the genes (*Lpl*, *Fabp4*, *Dgat1*, *Adipoq*, and *Glut4*) were lower in TNF-α-treated cells than in BSA-treated cells. Treatment with butyric acid (*Lpl*, *Dgat1*, *Dgat2, Adipoq*, and *Pparg2*), caprylic acid (*Fabp4*, *Dgat1*, and *Adipoq*), and capric acid (*Fabp4* and *Dgat1*), but not palmitic acid induced the expression of these genes in TNF-α-treated cells (Fig. 1B). The qRT-PCR data for genes with increased expressions after administration of TNF-α are shown in Supplementary Fig. S3.

**Fig. 2:**
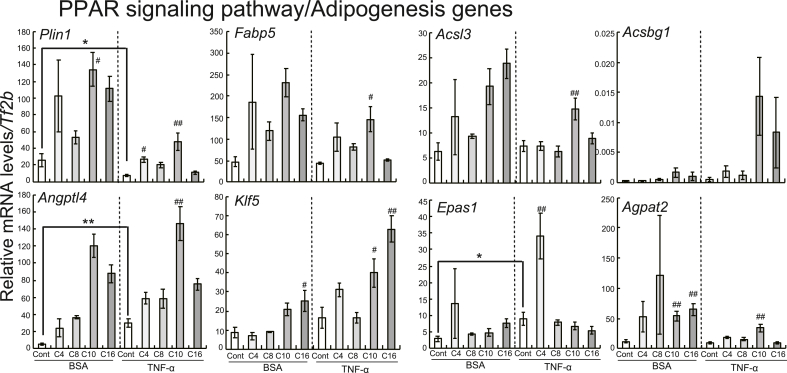
Effects of treatment with fatty acids (1000 μM) on the expressions of genes related to the Adar 1 editing deficiency immune response pathway in 3T3-L1 adipocytes with and without TNF-α administration. After reaching 80% confluence, 3T3-L1 cells were treated with adipocyte differentiation media for 96 h (regarded as day 0) and subsequently cultured in 10% FBS-containing DMEM for 48 h. The cells were incubated with or without TNF-α (BSA only) and individual fatty acids (butyric acid [C4], caprylic acid [C8], capric acid [C10], or palmitic acid [C16]) for 2 d qRT-PCR was performed, with target mRNA levels normalized using *Tf2b* mRNA levels. The data are represented as the means ± SEM for the 6 plates. Statistical analyses for differences between two groups (BSA-Cont and T-Cont cells) were performed using Student's *t*-test (*P < 0.05, **P < 0.01). Statistical analyses for differences among three or more groups treated with fatty acids were carried out using Dunnett's test based on ANOVA (^#^*P* < 0.05, ^##^*P* < 0.01).

Next, we performed pathway analysis on microarray data using wikiPathways. Genes associated with osteoclasts (*Dusp1*, *Saa 3,* and *Cxcl5*) and the chemokine signaling pathway (*Ccl2*, *Ccl7,* and *Cxcl5*) were upregulated in the TNF-α treated cells compared with the DMSO-treated cells (Supplementary Table S3). We found that nine upregulated genes associated with the PPAR signaling pathway and eight downregulated and two upregulated genes associated with the Focal Adhesion-PI3K-Akt-mTOR-signaling pathway were observed in the cells treated with capric acid compared with those treated with DMSO among the TNF-α-treated groups (Supplementary Table S4). Among the PPAR signaling pathway and adipogenesis genes, the expressions of *Plin1*, *Fabp5*, *Acsl3*, *Angptl4*, *Klf5,* and *Agpat2*, but not *Acsbg1*, *Epas1,* and *Acsbg1*, were enhanced by capric acid in the TNF-α-treated group. Expressions of these genes, except *Klf5* and *Epas1,* were higher in the TNF-α treated cells administered capric acid compared with those administered other fatty acids (Fig. 2). With respect to the focal adhesion-PI3K-Akt-mTOR-signaling pathway, expression of *Csf3* was higher in the DMSO-treated cells than in the BSA-treated cells among the TNF-α-treated groups. Furthermore, treatment with fatty acids in the TNF-α-treated groups enhanced the expression of *Csf3* (Fig. S4)*.* In addition, one upregulated and 25 downregulated genes associated with the Adar1 editing deficiency immune response were found in the cells treated with butyric acid compared with those treated with DMSO (Supplementary Table S5), and most of these expressional changes were confirmed by qRT-PCR (Fig. S5).

The mRNA expression of ATP citrate lyase (*Acly*) did not decrease upon TNF-α treatment, and *Acly* mRNA was induced by capric acid and palmitic acid in 3T3-L1 adipocytes without, but not with TNF-α. The mRNA expression of acetyl-CoA synthase 2 (*Acss2*) tended to be reduced by TNF-α treatment (*P* = 0.093), and capric acid and palmitic acid significantly induced these mRNA levels in 3T3-L1 adipocytes without TNF-α. In TNF-α treated adipocytes, capric acid was observed to induce this mRNA expression (Fig. S6).

### 3.2 Acetylation of histones H3 and H4 around genes induced by fatty acids in 3T3-L1 adipocytes

**Fig. 3:**
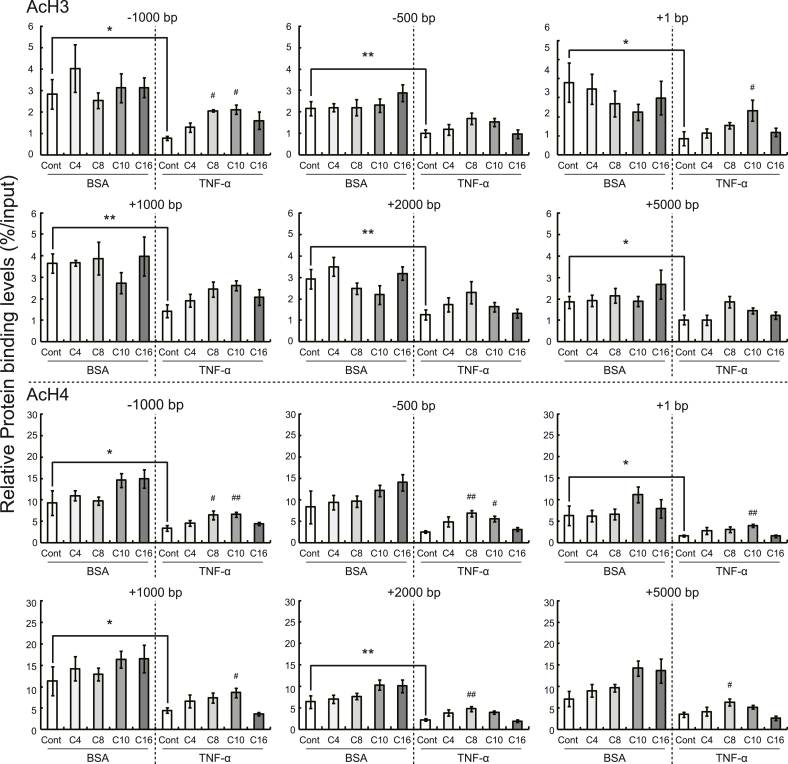
Effects of treatment with fatty acids (1000 μM) on the acetylation of histones around *Cidec* in 3T3-L1 adipocytes with and without TNF-α administration. After reaching 80% confluence, 3T3-L1 cells were treated with adipocyte differentiation media for 96 h (regarded as day 0) and subsequently cultured with 10% FBS-containing DMEM for 6 d. The cells were incubated with or without TNF-α (BSA only) and individual fatty acids (butyric acid [C4], caprylic acid [C8], capric acid [C10], or palmitic acid [C16]), for 48 d. ChIP signals for acetylated histones H3 and H4 were detected using qRT-PCR and normalized using the input signals. The data are represented as the means ± SEM for the 6 plates. Statistical analyses for differences between two groups (BSA-Cont and T-Cont cells) were performed using Student's *t*-test (*P < 0.05, **P < 0.01). Statistical analyses for differences among three or more groups treated with fatty acids were carried out using Dunnett's test based on ANOVA (^#^*P* < 0.05, ^##^*P* < 0.01).

**Fig. 4:**
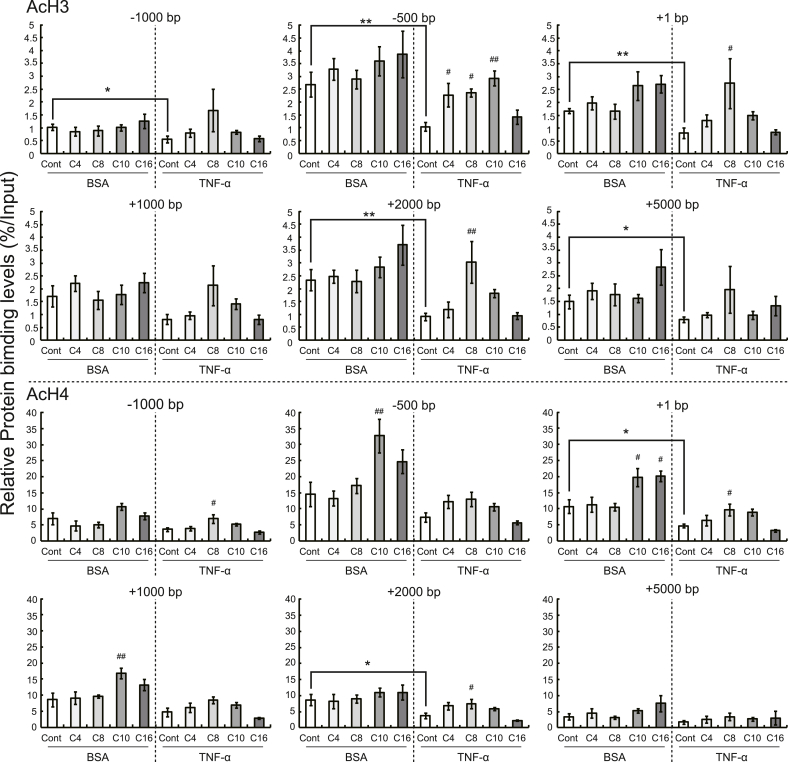
Effects of treatment with fatty acids (1000 μM) on the acetylation of histones around *Gpd1* in 3T3-L1 adipocytes with and without TNF-α administration. After reaching 80% confluence, 3T3-L1 cells were treated with adipocyte differentiation media for 96 h (regarded as day 0) and subsequently cultured with 10% FBS-containing DMEM for 6 d. The cells were incubated with or without TNF-α (BSA only) and individual fatty acids (butyric acid [C4], caprylic acid [C8], capric acid [C10], or palmitic acid [C16]) for 48 h. ChIP signals for acetylated histones H3 and H4 were detected using qRT-PCR and normalized using the input signals. The data are represented as the means ± SEM for the 6 plates. Statistical analyses for differences between two groups (BSA-Cont and T-Cont cells) were performed using Student's *t*-test (*P < 0.05, **P < 0.01). Statistical analyses for differences among three or more groups treated with fatty acids were carried out using Dunnett's test based on ANOVA (^#^*P* < 0.05, ^##^*P* < 0.01).

Next, we examined the acetylation levels of histones H3 and H4 around *Gpd1* and *Cidec*. These are the genes observed through microarray analysis and subsequent qRT-PCR analysis to be the most upregulated by short- and/or medium-chain fatty acids in 3T3-L1 adipocytes treated with TNF-α, using ChIP assays. The acetylation levels of histones H3 and H4 around *Cidec* and *Gpd1* in the promoter and gene body regions were lower in the cells administered TNF-α than in those administered BSA. Additionally, the acetylation levels in the cells treated with TNF-α were higher in the cells treated with short- and medium-chain fatty acids than in those treated with DMSO. However, medium-chain fatty acids induced higher levels of acetylation of histones H3 and H4 in the promoter and gene body regions compared with short-chain fatty acids (Fig. 3, Fig. 4).

The PPARG binding signals around the genes were not changed by co-administration of each fatty acid with TNF-α (Supplementary Fig. S7). Furthermore, the average of ChIP signals for IgG for all genes were 0.170% (*Cidec*) and 0.172% (*Gpd1*). Each ChIP signal for IgG around *Cidec* and *Gpd1* was shown in Supplementary Fig. S8.

## 4 Discussion

In this study, we found using microarray and subsequent qRT-PCR analyses that the expressions of lipid metabolic genes, such as *Cidec, Gpd1,* and *Cyp4b1*, decreased upon TNF-α treatment and increased upon treatment with butyric acid (*Gpd1*), caprylic acid (*Cidec* and *Gpd1*), and/or capric acid (*Cidec, Gpd1* and *Cyp4b1*) in TNF-α treated 3T3-L1 adipocytes. In addition, we found that the expression of many typical lipid metabolism-related genes, such as *Lpl*, *Fabp4, Dgat1*, and *Adipoq,* decreased upon TNF-α treatment and increased upon treatment with butyric acid (*Lpl, Dgat1,* and *Adipoq*), caprylic acid (*Fabp4, Dgat1,* and *Adipoq*), and/or capric acid (*Fabp4, Dgat1,* and *Adipoq*), but not palmitic acid in TNF-α treated 3T3-L1 adipocytes. This included several genes, such as *Lpl* involved to incorporate fatty acid into the adipocytes and re-synthesized into the triacylglycerol by digesting triacylglycerol into fatty acids and glycerol [20], *Fabp4* involved in fatty acid binding in cytoplasm [21], *Dgat1* involved in triglyceride synthesis [22], *Gpd1* involved in glycerol triphosphate synthesis [23], *Cidec* involved in lipid droplet formation [24], solute carrier family 2 (facilitated glucose transporter) member 4 (*Glut4*) [25], and *Adipoq* involved in insulin sensitivity of other tissues such as liver skeletal muscle [6]. *Adipoq*, *Lpl*, *Dgat1*, and *Fabp4* are well known PPARG target genes [5,26,27]. *Cidec* and *Gpd1* are PPARG target genes as well, and these genes are crucial for triglyceride accumulation [28,29]. These findings indicate that administration of medium- and short-chain fatty acids, but not long-chain fatty acids, restore the expression levels of the genes related to lipid metabolism, which are target genes for PPARG and were downregulated by TNF-α in adipocytes.

Among PPARG-target lipid metabolism-genes upregulated by medium- and short-chain fatty acids, we examined histone acetylation around *Cidec* and *Gpd1*, which showed the highest upregulation in microarray analysis and in subsequent qRT-PCR analysis. We examined histone acetylation because our recent studies demonstrated that histone acetylation around lipid metabolism- and insulin sensitivity-genes including *Cidec*, and *Gpd1* in 3T3-L1 adipocytes were regulated by histone acetylation in 3T3-L1 adipocytes [30,31]. Interestingly, histone acetylation around *Cidec* and *Gpd1* was decreased by treatment with TNF-α and restored by co-administration with short-and medium-chain fatty acids. Furthermore, a higher degree of histone acetylation recovery was observed upon co-administration with medium-chain fatty acids compared with short-chain fatty acids. Meanwhile, histone acetylation recovery was not observed upon co-administration with a long-chain fatty acid. It remains unclear how histone acetylation around lipid metabolism-related genes was enhanced by co-administration of a short-chain fatty acid or medium-chain fatty acids. However, butyric acid and β-hydroxybutyric acid, a medium-chain fatty acid metabolite, have been found to demonstrate HDACi properties [15,32]. Furthermore, expression of *Adipoq* was reported to be induced by HDACi [33]. A High-fat diet has been reported to lower the expression of *Acly*, which encodes the enzyme that converts citrate to acetyl-CoA in cytosol, and fatty acid synthesis related genes such as fatty acid synthase (*Fasn*) in white adipose tissues in C57BL/6 J mice, the decrease of *Acly* expression level in adipocytes being caused by genetic deletion-induced global histone acetylation [34]. The decreased expression of *Acss2*, which encodes an enzyme that converts acetic acid to acetyl-CoA in cytosol, also reduced histone acetylation around downregulated genes in a neuronal cell culture model [35]. Previous studies have also shown that intake of medium-chain fatty acids enhanced the production of pyruvic acid and ketone bodies [16]. In this study, we demonstrated that TNF-α treatment tended to reduce *Acss2* (*P* = 0.093) mRNA expression and capric acid significantly increased these mRNA levels in TNF-α treated adipocytes. Thus, reduction and induction of histone acetylation by TNF-α and medium- and short-chain fatty acids, respectively, may be caused by decreases or increases in acetyl-CoA and ketone bodies. Notably, we attempted to determine the cellular amounts of acetyl-CoA and β-hydroxybutyric acid using ELISA method and enzymatic assay, respectively, but were unable to measure acetyl-CoA and β-hydroxybutyric acid levels (data not shown), perhaps due to the quick metabolism and the low stabilities of acetyl-CoA and β-hydroxybutyric acid. Future studies are required to identify the metabolites produced after treatment with medium- or short-chain fatty acids in adipocytes, including acetyl-CoA and β-hydroxybutyric acid. Furthermore, the effect of these metabolites on histone acetylation around metabolic genes in adipocytes needs to be measured using highly sensitive methods.

The contribution of the acetylation of each lysine residue of histones on the expression of lipid-metabolism related genes in 3T3-L1 adipocytes co-treated with TNF-α and medium chain fatty acids remains unclear. We chose pan-acetyl antibodies and amplified a broad range of regions because our previous studies have demonstrated that pan-acetylation of histone around adipocyte genes, such as *Adipoq* and *Lpl,* increased during adipocyte differentiation and decreased upon TNF-α treatment [6,8]. Furthermore, these genes are regulated by the acetylated histone reader bromodomain-containing protein 4 (BRD4) [31], which strongly binds to di-acetylated histone H3 at lysine 9 and 14 and tetra-acetylated histone H4 at lysine 5, 8, 12, 16, rather than individual acetylated lysine [36]. However, it has been reported that histone H3K9 and K27 acetylation is important for transcription activation and repression because these lysine residues of histone H3 are also methylated and induces transrepression [37]. Further studies are required to investigate whether acetylation and methylation of histone H3 at each lysine are altered by TNF-α treatment with/without short-, medium-, and long-chain fatty acids in 3T3-L1 adipocytes.

Previous studies have demonstrated that histone acetylation not only induces euchromatin formation from heterochromatin, but also recruits transcription initiation and elongation complexes to the promoter/enhancer and gene body regions, respectively [38,39]. In this study, medium- and short-chain fatty acids induced histone acetylation in these regions around lipid metabolism-related genes in adipocytes. Therefore, medium- and short-chain fatty acid may enhance transcription initiation and elongation reactions. However, this hypothesis requires confirmation in further studies.

A previous study showed using luciferase assays that the responsive elements of PPARG2 in the adipocytes were located within −1000 to +1 bp upstream of *Cidec* [40]. Furthermore, treatment using insulin and indomethacin, a PPAR activator, induced the expression of *Gpd1* in adipocytes [41]. However, we demonstrated that TNF-α treatment did not reduce *Pparg2* expression and PPARG binding around *Cidec* and *Gpd1* in 3T3-L1 adipocytes. Additionally, the PPAR signals around *Gpd1* were higher than the IgG signals. Therefore, histone acetylation, around *Gpd1* may affect its expression in 3T3-L1 adipocytes treated with TNF-α and fatty acids. On the other hand, the PPARG signals around *Cidec* were not higher than IgG signals. Therefore, further investigation using sensitive ChIP assays on whether PPARG is bound to the upstream region of *Cidec* in the 3T3-L1 adipocytes are required. Additionally, the reduced enhancement of PPARG located upstream of *Gpd1* and *Cidec* by TNF-α in 3T3-L1 adipocytes as well as the effect of fatty acids on them require further investigation.

In this study, we found that TNF-α treatment induced several genes related to the Adar1 editing deficiency immune response, which involves interferon signals activated by double strand RNA [42]. We found that the expressions of these genes were reduced by butyric acid as well as caprylic acid and capric acid to a lesser degree. These results indicate that butyric acid reduces inflammation triggered by TNF-α treatment. It should be noted that palmitic acid and butyric acid demonstrated similar reductions in inflammation-related gene expressions in the TNF-α treated cells. A recent study demonstrated that intake of long-chain saturated fats was associated with increased risk of coronary heart disease development [43]. In contrast, we demonstrated that palmitic acid reduced expressions of insulin sensitivity genes, such as *Lpl* and *Pparg2,* in TNF-α treated adipocytes. However, butyric acid, caprylic acid, and capric acid enhanced the expressions of several insulin sensitivity genes. Therefore, TNF-α and palmitic acid may affect different inflammation signals in adipocytes. Further exploration of the different inflammatory signals are required.

We also found that capric acid enhanced the PPAR signaling pathway and the expressions of adipogenesis genes, including *Plin1*, *Fabp5*, *Acsl3*, *Abgptl4,* and *Agpat2*. This induction was stronger than those caused by butyric acid or caprylic acid. We also demonstrated that the induction of the expressions of insulin sensitivity genes, such as *Fabp4*, *Dgat1*, *Cyp4b1*, *Cidec*, and *Glut4*, in TNF-α treated adipocytes were greater in cells treated with capric acid compared with those treated with butyric acid and caprylic acid. Therefore, capric acid may have higher efficacy of insulin resistance than butyric acid and caprylic acid. Additionally, triglycerides composed of capric acid ameliorated myocardial abnormalities in mice with triglyceride deposit cardiomyovasculopathy [44]. However, triglycerides containing caprylic acid have not yet been compared with those containing capric acid. Further studies should examine the effect of triglycerides containing capric acid on lipid abnormalities, including insulin resistance, in adipose tissues of animals. However, cell viabilities were shown to be reduced by capric acid at concentrations of 200–100 μM, indicating that its safety profile in animals with insulin resistance should also be investigated.

It is still unclear whether protein expression of *Cidec* and *Gpd1* is altered by TNF-α and/or fatty acids in 3T3-L1 adipocytes. In addition, it still remains unclear whether other histone modifications around lipid metabolism and/or PPARG target genes are altered by short- and/or medium chain fatty acid treatment in 3T3-L1 adipocytes, although our previous studies have demonstrated that TNF-α treatment reduces histone acetylation around *Adipoq* and *Lpl* [6,8]. These topics remain to be examined in further works.

In conclusion, we demonstrated that the administration of medium- and short-chain fatty acids can restore the reduced expression of *Cidec* and *Gpd1*, which are genes related to lipid metabolism, by promoting histone acetylation around these genes in TNF-α-treated insulin-resistant adipocytes.

## Funding

The work presented in this article was supported by the KAKENHI program of the 10.13039/501100001691Japan Society for the Promotion of Science (JP20H04103, JP17H01964, JP20K21750) from the Ministry of Education, Culture, Sports, Science and Technology; the Takeda Science Foundation; and the Uehara Memorial Foundation.

## Author contributions

M. Kawamura performed most of the experiments and wrote the manuscript. N Goda, N. Hariya, M. Kimura, and S. Ishiyama helped perform the experiments. T. Kubota and K. Mochizuki helped draft the manuscript. K. Mochizuki organized the study. All authors have approved the final article.

## Declaration of competing interest

The authors declare that they have no conflicts of interest.
