# Supplementary material for: Medium-chain fatty acids enhance expression and histone acetylation of genes related to lipid metabolism in insulin-resistant adipocytes
Source: Biochem Biophys Rep. 2022 Jan 5;29:101196. doi: 10.1016/j.bbrep.2021.101196 (PMC8741418; doi:10.1016/j.bbrep.2021.101196)
Supplement: Multimedia component 1 [file mmc1.pdf]

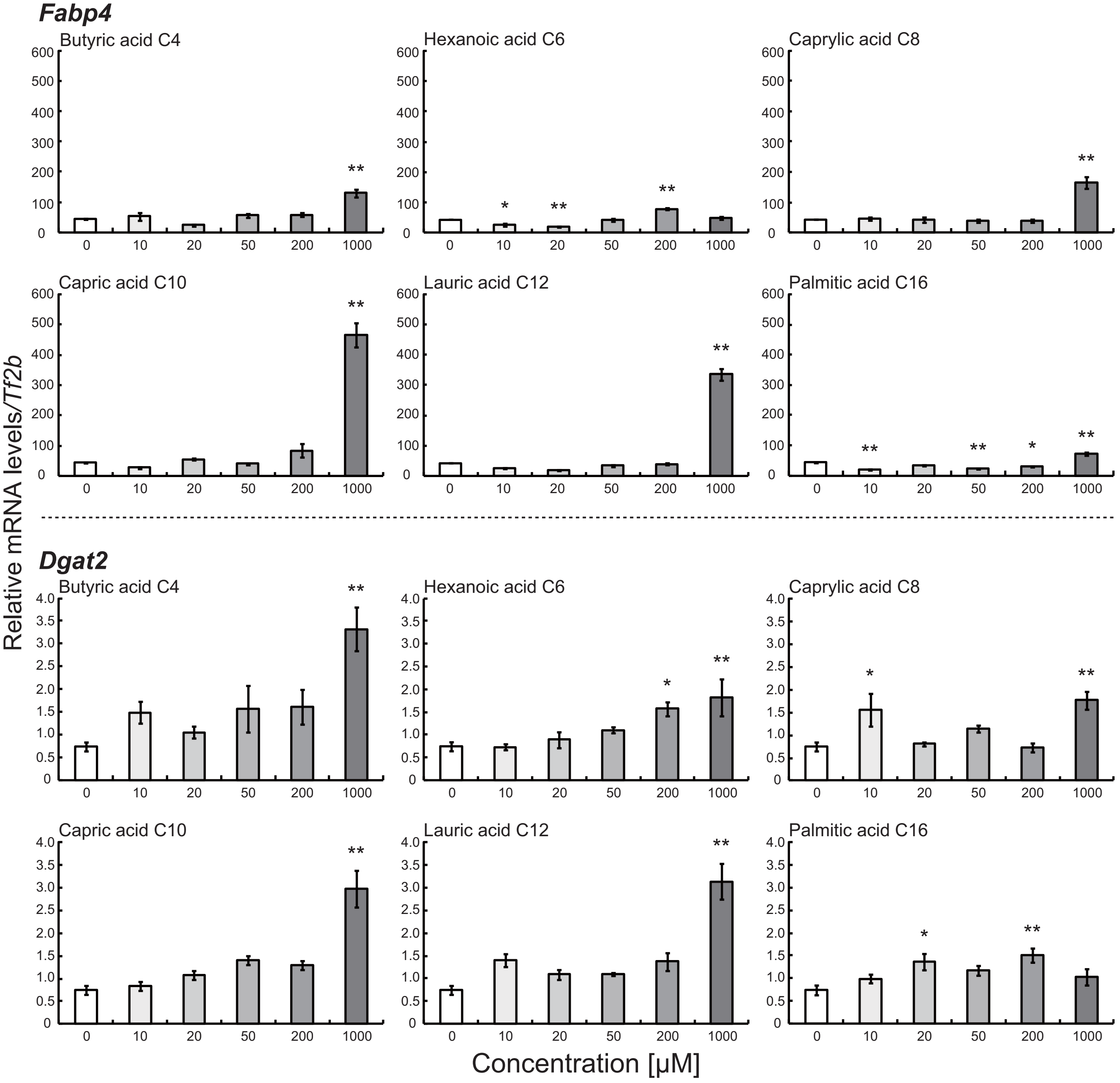

Fabp4
Butyric acid C4
Hexanoic acid C6
Caprylic acid C8
Capric acid C10
Lauric acid C12
Palmitic acid C16
Dgat2
Relative mRNA levels/Tf2b
Concentration [μM]
0
10
20
50
200
1000

Fig S1

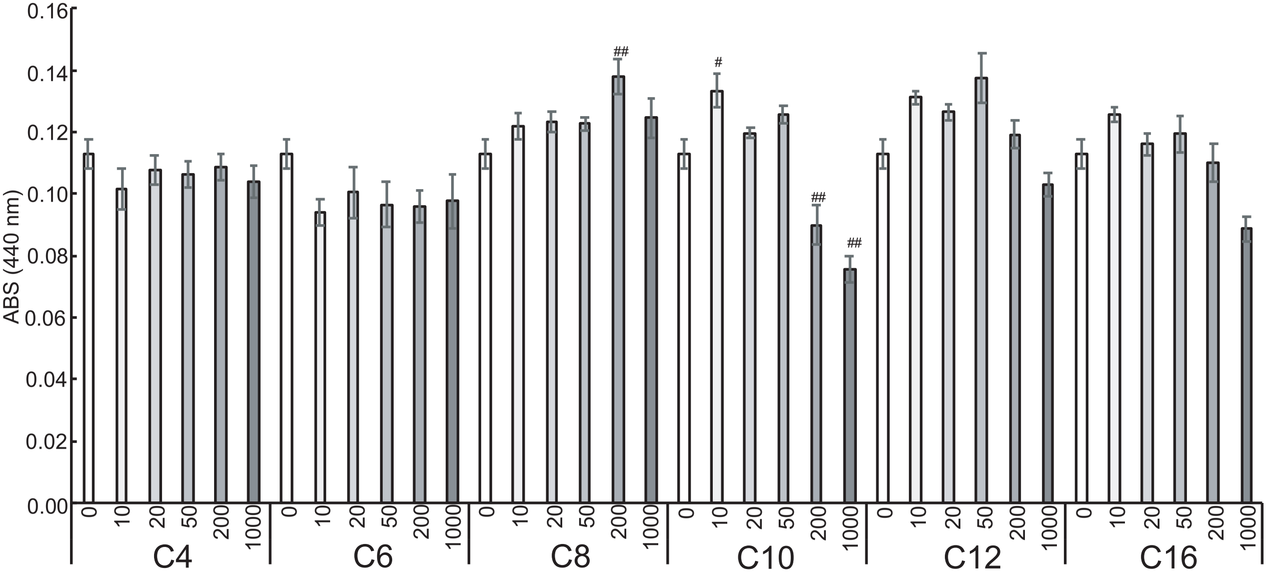
ABS (440 nm)
0.00
0.02
0.04
0.06
0.08
0.10
0.12
0.14
0.16
##
#
##
##
0
10
20
50
200
1000
C4
C6
C8
C10
C12
C16

Fig S2

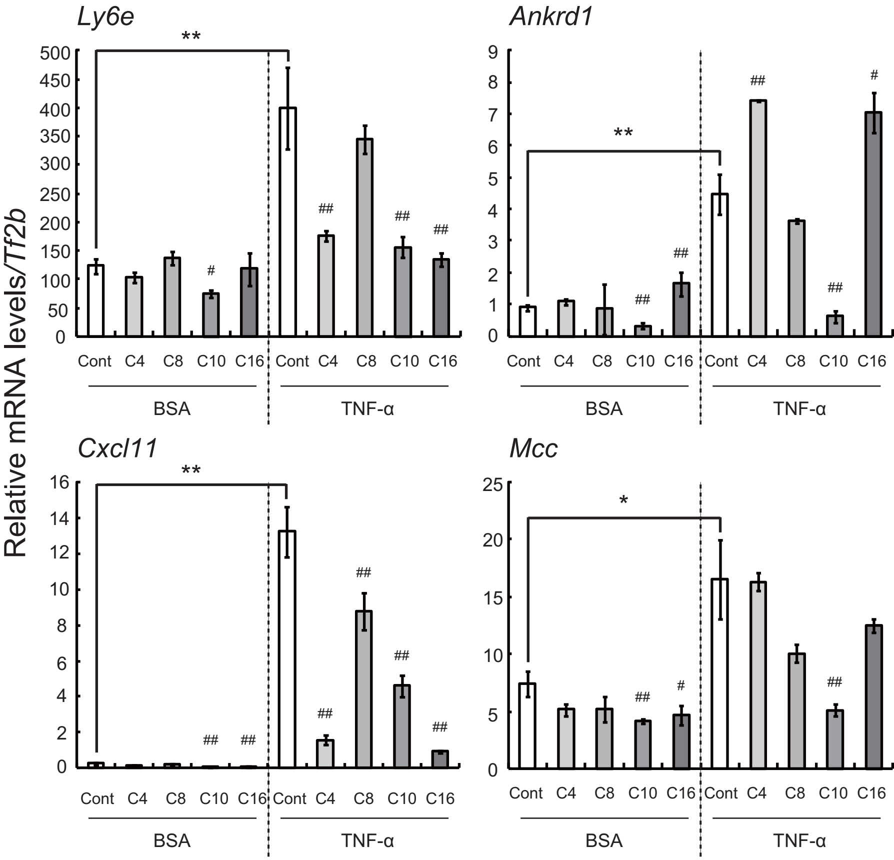
Ly6e
Ankrd1
Cxcl11
Mcc
Relative mRNA levels/Tf2b
Cont C4 C8 C10 C16 Cont C4 C8 C10 C16
BSA
TNF-α
**
*
#
##

Fig. S3

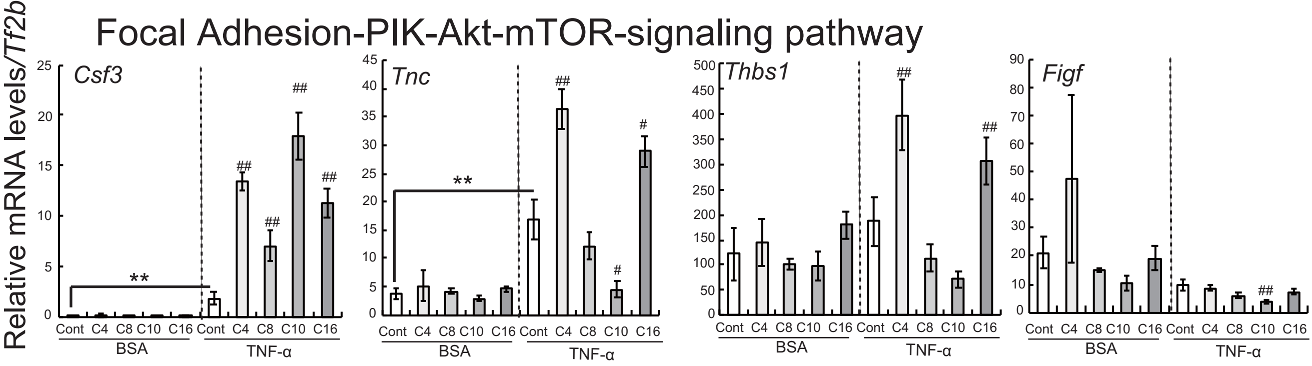
Focal Adhesion-PIK-Akt-mTOR-signaling pathway
Relative mRNA levels/Tf2b
Csf3
Tnc
Thbs1
Figf
**
##
#
Cont C4 C8 C10 C16 Cont C4 C8 C10 C16
BSA
TNF-α

Fig.S4

Adar 1 editing deficiency immune response

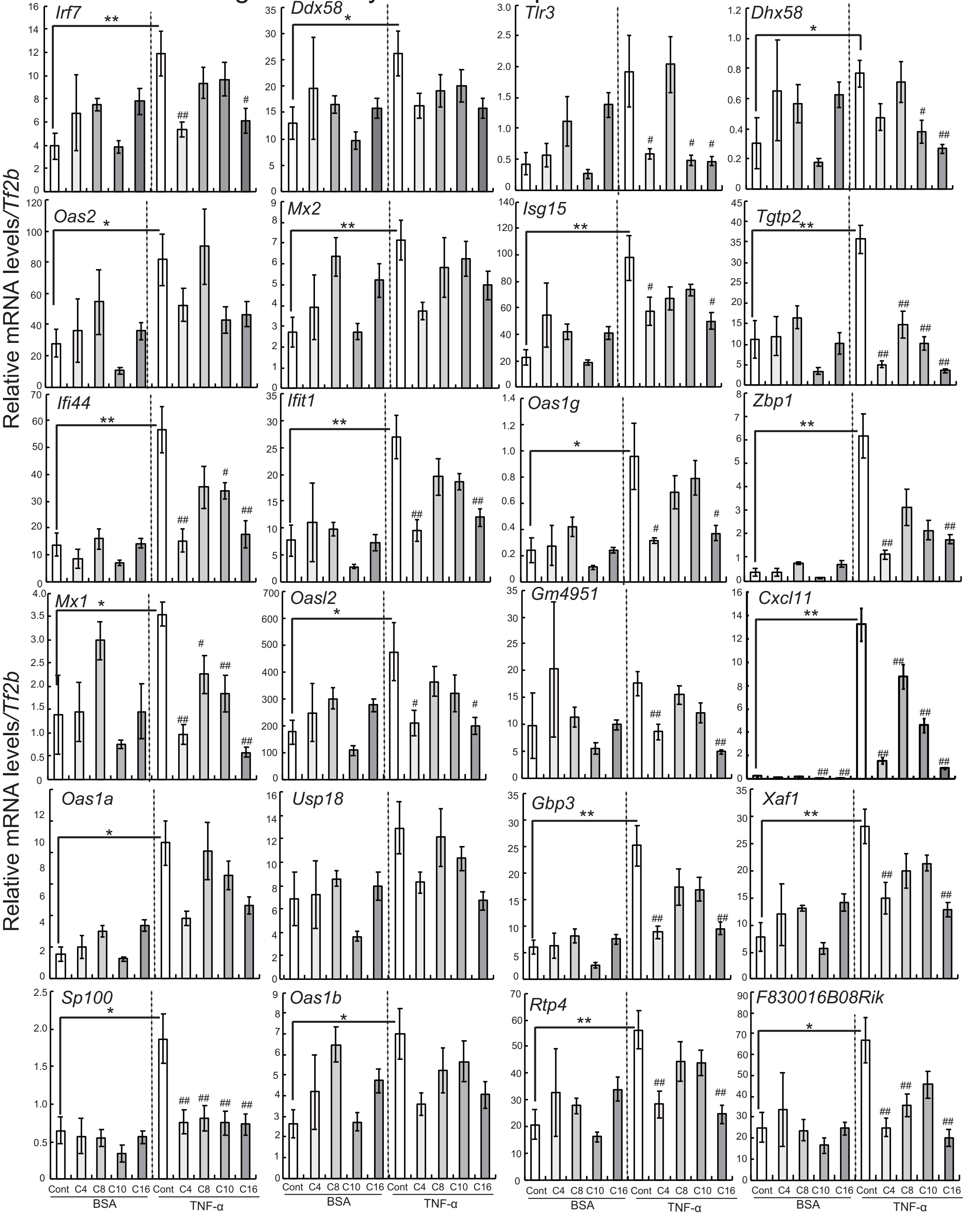

Fig.S5

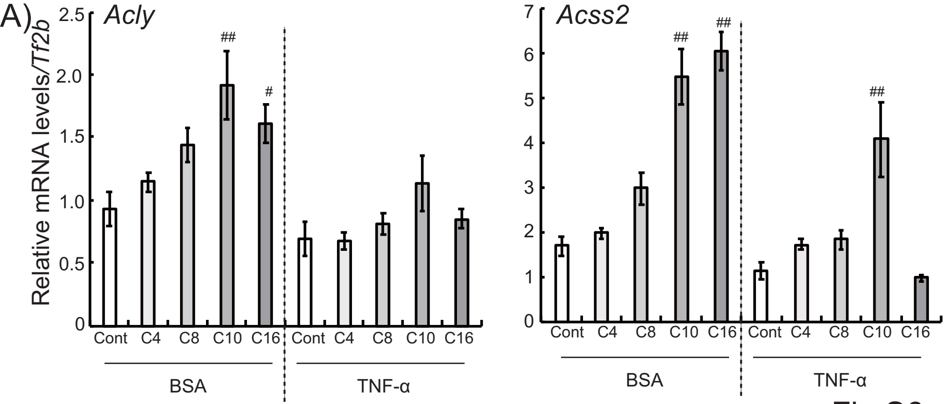
A)
Acly
Relative mRNA levels/Tf2b
2.5
2.0
1.5
1.0
0.5
0
##
#
Cont C4 C8 C10 C16 Cont C4 C8 C10 C16
BSA
TNF-α
Acss2
7
6
5
4
3
2
1
0
##
##
##
Cont C4 C8 C10 C16 Cont C4 C8 C10 C16
BSA
TNF-α

Fig.S6

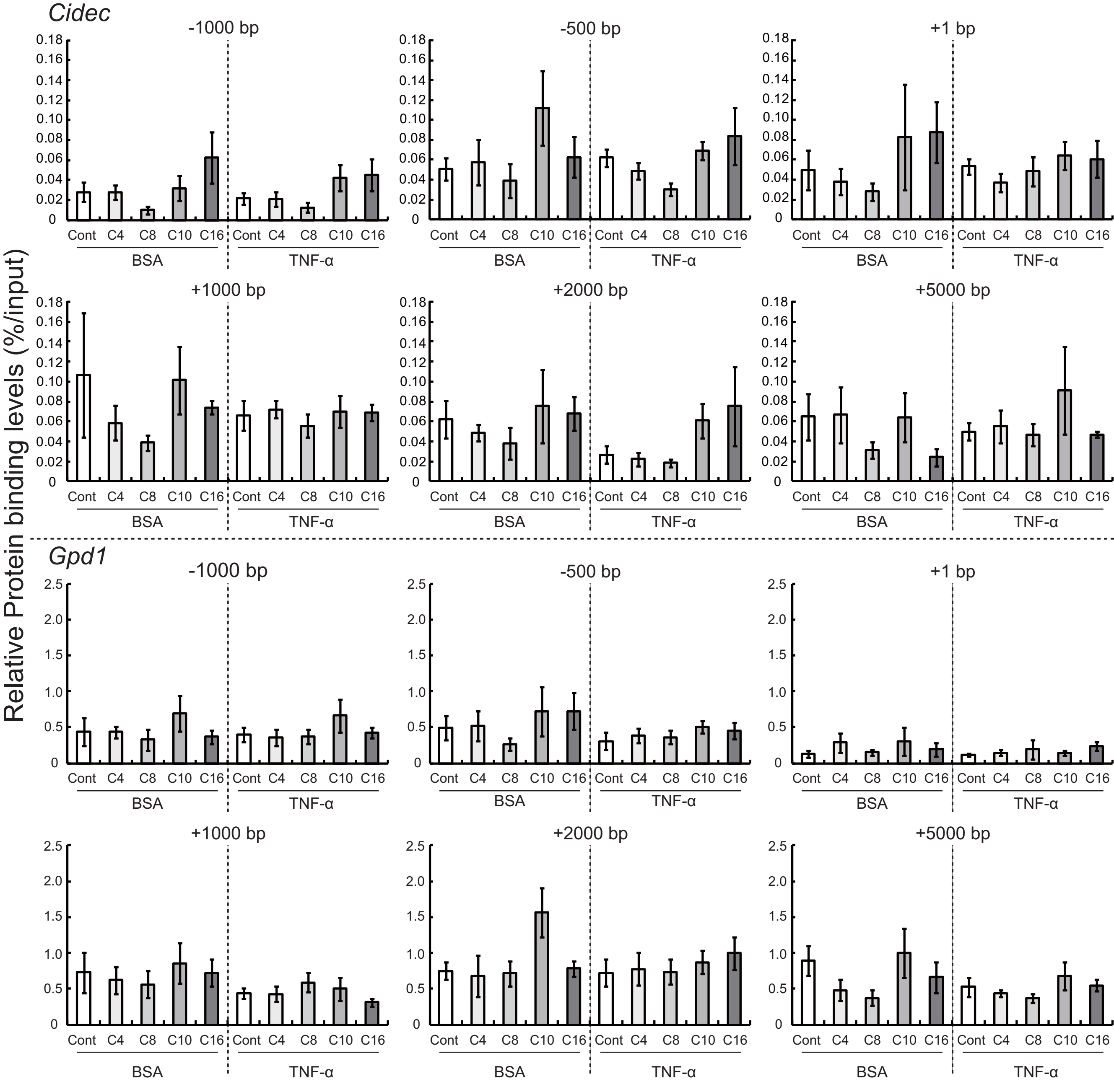

Cidec
-1000 bp
-500 bp
+1 bp
+1000 bp
+2000 bp
+5000 bp
Gpd1
-1000 bp
-500 bp
+1 bp
+1000 bp
+2000 bp
+5000 bp
Relative Protein binding levels (%/input)
0.18
0.16
0.14
0.12
0.10
0.08
0.06
0.04
0.02
0
2.5
2.0
1.5
1.0
0.5
0
Cont
C4
C8
C10
C16
BSA
TNF-α

Fig. S7

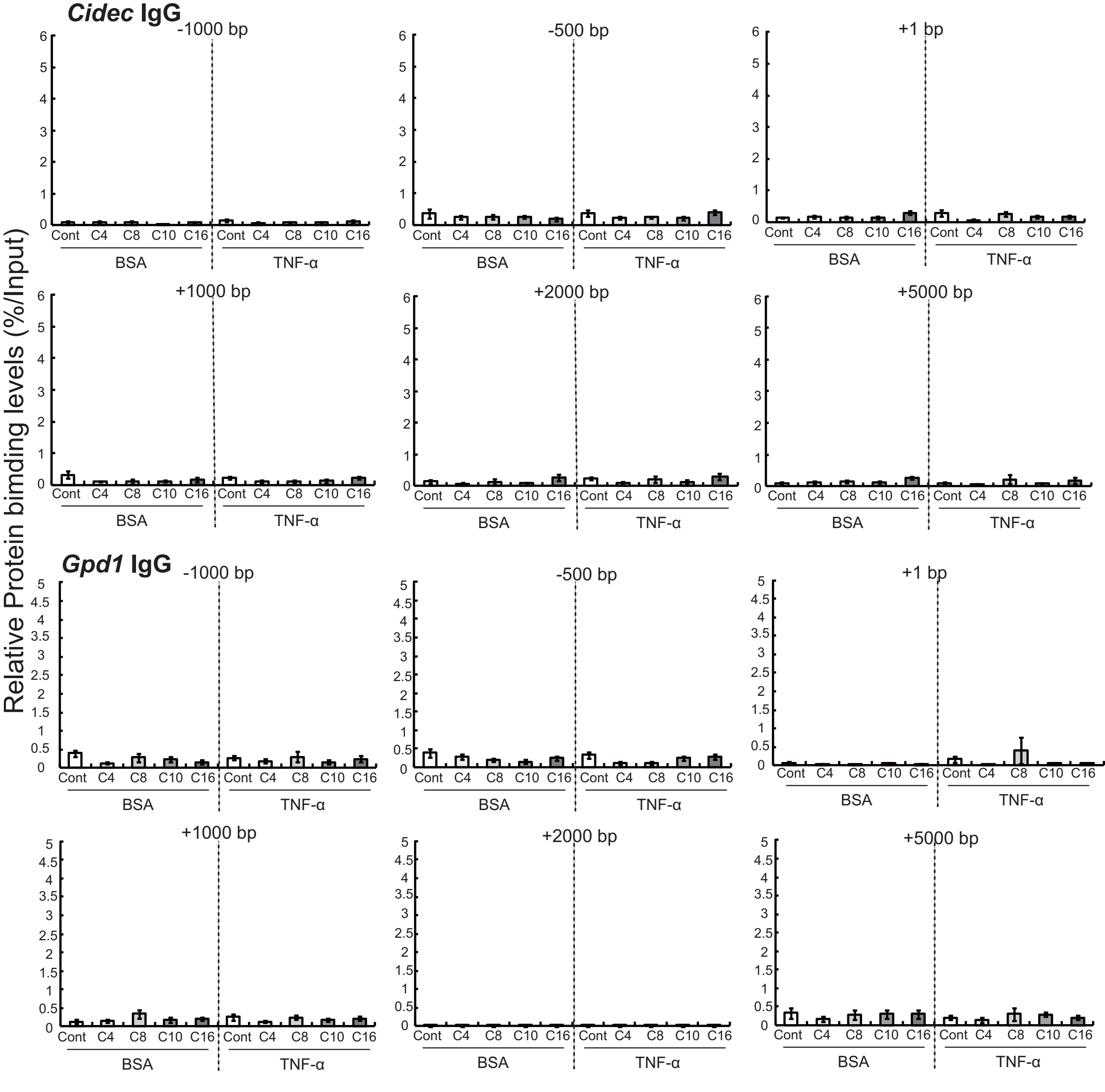
Cidec IgG
Gpd1 IgG
Relative Protein bimding levels (%/Input)
-1000 bp
-500 bp
+1 bp
+1000 bp
+2000 bp
+5000 bp
Cont
C4
C8
C10
C16
BSA
TNF-α

Fig. S8

Supplementary Figure legends

Supplementary Figure S1. Effects of treatment with various concentrations (0–1000 μM) of fatty acids on the expressions of *Fabp4* and *Dgat2* in 3T3-L1 adipocytes. After reaching 80% confluence, 3T3-L1 cells were treated with adipocyte differentiation media for 48 h (regarded as day 0) and subsequently cultured with 10% FBS-containing DMEM for 4 d. The cells were incubated with or without individual fatty acids (butyric acid [C4], hexanoic acid [C6], caprylic acid [C8], capric acid [C10], lauric acid [C12], or palmitic acid [C16]) at various concentrations (0, 10, 20, 50, 200, and 1000 μM) for 48 h. qRT-PCR was performed, with target mRNA levels normalized using *Tf2b* mRNA levels. The data are represented as the means ± SEM for the 6 plates. Statistical analyses for differences among three or more groups treated with fatty acids were carried out using Dunnett's test based on ANOVA (*$P<0.05$, **$P<0.01$).

Supplementary Figure S2. Effects of treatment with various concentrations (0–1000 μM) of fatty acids on the cell viabilities of adipocytes. After reaching 80% confluence, 3T3-L1 cells were treated with adipocyte differentiation media for 48 h (regarded as day 0) and subsequently cultured with 10% FBS-containing DMEM for 4 d. The cells were incubated with or without individual fatty acids (butyric acid [C4], hexanoic acid [C6], caprylic acid [C8], capric acid [C10], lauric acid [C12], or palmitic acid [C16]) at various concentrations (0, 10, 20, 50, 200, and 1000 μM) for 48 h. MTT assay was performed to assess cell viabilities. The data are represented as the means ± SEM for the 6 plates. Statistical analyses for differences among three or more groups

treated with fatty acids were carried out using Dunnett's test based on ANOVA (*P<0.05, **P<0.01).

Supplementary Figure S3. Effects of treatment with fatty acids (1000 μM) on the expressions of genes upregulated by TNF-α in the microarray analyses of 3T3-L1 adipocytes with and without TNF-α administration. After reaching 80% confluence, 3T3-L1 cells were treated with adipocyte differentiation media for 96 h (regarded as day 0) and subsequently cultured with 10% FBS-containing DMEM for 6 d. The cells were incubated with or without TNF-α (BSA only) and individual fatty acids (butyric acid (C4), caprylic acid (C8), capric acid (C10), or palmitic acid [C16]) for 48 h. qRT-PCR was performed, with target mRNA levels normalized using *Tf2b* mRNA levels. The data are represented as the means ± SEM for the 6 plates. Statistical analyses for differences between two groups (BSA-Cont and T-Cont cells) were performed using Student's *t*-test (*P<0.05, **P<0.01). Statistical analyses for differences among three or more groups treated with fatty acids were carried out using Dunnett's test based on ANOVA ($^{\#}P$<0.05, $^{\#\#}P$<0.01).

Supplementary Figure S4. Effects of treatment with fatty acids (1000 μM) on the expressions of genes related to the Focal Adhesion-PIK-Akt-mTOR-signaling pathway in 3T3-L1 adipocytes with and without TNF-α administration. After reaching 80% confluence, 3T3-L1 cells were treated with adipocyte differentiation media for 96 h (regarded as day 0) and subsequently cultured in 10% FBS-containing DMEM for 6 d. The cells were incubated with or without TNF-α (BSA only) and

individual fatty acids (butyric acid [C4], caprylic acid [C8], capric acid [C10], or palmitic acid [C16]) for 48 h. qRT-PCR was performed, with the target mRNA levels normalized using *Tf2b* mRNA levels. The data are represented as the means ± SEM for the 6 plates. Statistical analyses for differences between two groups (BSA-Cont and T-Cont cells) were performed using Student's *t*-test (**P*<0.05, ***P*<0.01). Statistical analyses for differences among three or more groups treated with fatty acids were carried out using Dunnett's test based on ANOVA ($^{\#}P<0.05$, $^{\#\#}P<0.01$).

Supplementary Figure S5. Effects of treatment with fatty acids (1000 μM) on the expressions of genes related to the Adar1 editing deficiency immune response pathway in 3T3-L1 adipocytes with and without TNF-α administration. After reaching 80% confluence, 3T3-L1 cells were treated with adipocyte differentiation media for 96 h (regarded as day 0) and subsequently cultured in 10% FBS-containing DMEM for 6 d. The cells were incubated with or without TNF-α (BSA only) and individual fatty acids (butyric acid [C4], caprylic acid [C8], capric acid [C10], or palmitic acid [C16]) for 48 h. qRT-PCR was performed, with the target mRNA levels normalized using *Tf2b* mRNA levels. The data are represented as the means ± SEM for the 6 plates. Statistical analyses for differences between two groups (BSA-Cont and T-Cont cells) were performed using Student's *t*-test (**P*<0.05, ***P*<0.01). Statistical analyses for differences among three or more groups treated with fatty acids were carried out using Dunnett's test based on ANOVA ($^{\#}P<0.05$, $^{\#\#}P<0.01$).

Supplementary Figure S6 Effects of treatment with fatty acids (1000 μM) on the

expressions of genes related to acetyl-CoA production in 3T3-L1 adipocytes with and without TNF-α administration. After reaching 80% confluence, 3T3-L1 cells were treated with adipocyte differentiation media for 96 h (regarded as day 0) and subsequently cultured with 10% FBS-containing DMEM for 6 d. The cells were incubated with or without TNF-α (BSA only) and individual fatty acids (butyric acid (C4), caprylic acid (C8), capric acid (C10), or palmitic acid [C16]) for 48 h. qRT-PCR was performed, with target mRNA levels normalized using *Tf2b* mRNA levels. The data are represented as the means ± SEM for the 6 plates. Statistical analyses for differences between two groups (BSA-Cont and T-Cont cells) were performed using Student's *t*-test (*P<0.05, **P<0.01). Statistical analyses for differences among three or more groups treated with fatty acids were carried out using Dunnett's test based on ANOVA ($^{\#}P$<0.05, $^{\#\#}P$<0.01).

Supplementary Figure S7. Effects of treatment with fatty acids (1000 μM) on PPARγ binding around the *Cidec* and *Gpd1* genes in 3T3-L1 adipocytes with and without TNF-α administration. After reaching 80% confluence, 3T3-L1 cells were treated with adipocyte differentiation media for 96 h (regarded as day 0) and subsequently cultured with 10% FBS-containing DMEM for 6 d. The cells were incubated with or without TNF-α (BSA only) and individual fatty acids (butyric acid [C4], caprylic acid [C8], capric acid [C10], or palmitic acid [C16]) for 48 h. The genomic DNA cross-linked to nuclear proteins in adipocytes were sonicated and precipitated using anti-PPARG antibodies. The primer pairs used for PCR analysis are listed in **Supplementary Table 2** and **Supplementary Table 3**. ChIP signals were

detected using qRT-PCR and normalized using the input signals. The data are represented as the means ± SEM for the 6 plates. Statistical analysis for differences between two groups (BSA-Cont and T-Cont cells) were performed using Student's *t*-test. Statistical analyses for differences among three or more groups treated with fatty acids were carried out using Dunnett's test based on ANOVA. Significant differences were not observed.

Supplementary Figure S8. Effects of treatment with fatty acids (1000 μM) on IgG binding around the *Cidec* and *Gpd1* genes in 3T3-L1 adipocytes with and without TNF-α administration. After reaching 80% confluence, 3T3-L1 cells were treated with adipocyte differentiation media for 96 h (regarded as day 0) and subsequently cultured with 10% FBS-containing DMEM for 6 d. The cells were incubated with or without TNF-α (BSA only) and individual fatty acids (butyric acid [C4], caprylic acid [C8], capric acid [C10], or palmitic acid [C16]) for 48 h. The genomic DNA cross-linked to nuclear proteins in adipocytes were sonicated and precipitated using anti-PPARG antibodies. The primer pairs used for PCR analysis are listed in **Supplementary Table 2** and **Supplementary Table 3**. ChIP signals were detected using qRT-PCR and normalized using the input signals. The data are represented as the means ± SEM for the 6 plates. Statistical analyses for differences between two groups (BSA-Cont and T-Cont cells) were performed using Student's *t*-test. Statistical analyses for differences among three or more groups treated with fatty acids were carried out using Dunnett's test based on ANOVA. Significant differences were not observed.
