# Supplementary material for: Medium-chain fatty acids enhance expression and histone acetylation of genes related to lipid metabolism in insulin-resistant adipocytes
Source: Biochem Biophys Rep. 2022 Jan 5;29:101196. doi: 10.1016/j.bbrep.2021.101196 (PMC8741418; doi:10.1016/j.bbrep.2021.101196)
Supplement: Multimedia component 2 [file mmc2.pdf]

Supplementary Table S1. Sequences of oligonucleptide primers used for qRT-PCR.

	Symbol	Sequence（5′-3′）
Transcription factor IIB	*Tf2b*	GACAGTCCTACAGACTGATCTACCC<br>TCCACTGGGGTGTCAAACTT
Lipoprotein lipase	*Lpl*	CTGGTGGGAAATGATGTGG<br>TGGACGTTGTCTAGGGGGTA
Fatty acid-binding protein (aP2, ALBP)	*Fabp4*	GGATGGAAAGTCGACCACAA<br>TGGAAGTCACGCCTTTCATA
Diacylglycerol O-acyltransferase 1	*Dgat1*	GCTTCTGCAGTTTGGAGACC<br>TTCTGCCAAAAGTAGGTGACAG
Diacylglycerol O-acyltransferase 2	*Dgat2*	TACTCCAAGCCCATCACCAC<br>GGCATGGTACAGGTCGATGT
Glycerol-3-phosphate dehydrogenase 1 (soluble)	*Gpd1*	ATTGAGCAGCTGGAGAAGGA<br>GCAGTGAACAAGGGGAACTT
Solute carrier family 2 (facilitated glucose transporter), member 4	*Glut4*	GGATACACCCTCCCAAAATCT<br>CCTTGCCCTGTCAGGTATGT
Adiponectin, C1Q, and collagen domain containing	*Adipoq*	GAATGTGGACCAGGCCTCT<br>ACCTGGAGCCAGACTTGGT
Family with sequence similarity 213, member A	*Fam213a*	GGAAACCTAGAAGGCGAAGG<br>CTCGGTGCTCAAGAAGGATG
Paternally expressed 10	*Peg10*	GGACCCCTCATCCTTCGT<br>TTCTTAAAACCCGCCTGTTC
Cytochrome P450, family 4, subfamily b, polypeptide 1	*Cyp4b1*	GCTCAGCAAGCCAGTAACCT<br>AGGGCATAGATGTGCAGAGAG
CD248 antigen, endosialin	*Cd248*	TTCCAGAGCCCCTCTAGTCC<br>AGCTGTGGCACTGATTTGG
Melanoma cell adhesion molecule	*Mcam*	TGCTGAATCTGTCTTGTGAGG<br>CTGTGGATCTGGGTTCCATT
Cell death-inducing DFFA-like effector c	*Cidec*	GAAGGTTCGCAAAGGCATC<br>CAGGGAGAAGGGCTTGTCTT
Peroxisome proliferator-activated receptor gamma 1	*Pparg1*	GAGGACGCGGAAGAAGAGACCT<br>CAGTGGTTCACCGCTTCTTTCA
Peroxisome proliferator-activated receptor gamma 2	*Pparg2*	GATTCTCCTGTTGACCCAGAGCA<br>CATAGGCAGTGCATCAGCGAAG
Lymphocyte antigen 6 complex, locus E	*Ly6e*	TCTGGGCATGGAGCAAGT<br>CTTTCTCCTGGCATGAAACTG

Ankyrin repeat domain 1 (cardiac muscle)	*Ankrd1*	CGGACCTCAAGGTCAAGAAC<br>TGAGGCTGTCGAATATTGCTT
Chemokine (C-X-C motif) ligand 11	*Cxcl11*	AAGCAAGCTCGCCTCATAAT<br>CAGGTTCCTGGCACAGAGTT
Mutated in colorectal cancers	*Mcc*	ATGTAAAAGCAACGCCGAGA<br>GGCTTCTATGCACTGCTCACT
Interferon Regulatory Factor 7	*Irf7*	CTTCAGCACTTTCTTCCGAGA<br>TGTAGTGTGGTGACCCTTGC
DExD/H-Box Helicase 58	*Ddx58*	AGACCACAAACTTGGAGAGTCAC<br>GCGGTCTTAGCATCTCCAAC
Toll-like receptor 3	*Tlr3*	GATACAGGGATTGCACCCATA<br>TCCCCCAAAGGAGTACATTAGA
DExH-Box Helicase 58	*Dhx58*	TGTGAACCCCAACTTCTCG<br>CCTTAAAGACCTTGTTAATGACCAC
2'-5'-Oligoadenylate Synthetase 2	*Oas2*	CTTCCAGTCTTCAATCCTCTACG<br>TGGTGAGGCTCTTACTTGATCC
MX Dynamin Like GTPase 2	*Mx2*	CAAGGAACACCCTCATTTCAG<br>AGATGAGCTCTGCGGTCAGT
Interferon-stimulated gene 15	*Isg15*	GAACAAGTCCACGAAGACCAG<br>GCAGCTCCTTGTCCTCCAT
T-cell-specific guanine nucleotide triphosphate-binding protein 2	*Tgtp2*	CAGATCAAGGTCACCACTGC<br>AGAGATGATTTTGCTTTCCCTTT
Interferon-induced protein 44-like	*Ifi44*	GCCAGCCTCGGTACAGACT<br>CAGTCTAGTGGAAAAGCCCTCT
Interferon-induced protein with tetratricopeptide repeats 1	*Ifit1*	ACTGATTCCTGCTGTTTTGGA<br>GGCACAGACATAAGGAAAAAGG
2'-5' oligoadenylate synthetase 1G	*Oas1g*	AGAGTCAAGTTTGAGGTCCAGAG<br>GGCTTCTTATTGATACTACCATGACC
Z-DNA-binding protein 1	*Zbp1*	CAGGAAGGCCAAGACATAGC<br>ACAAATAATCGCAGGGGACT
MX Dynamin Like GTPase 1	*Mx1*	GCACCTGAAAGCCTACTACCA<br>GGATGAAGTACTGGATAATCAGAGG
2'-5'-oligoadenylate synthase-like protein 2	*Oasl2*	GTTCTTTCGGGAACAGTGCT<br>GTTCAGCGTTGTCCCCTTT
Interferon-gamma-inducible GTPase Ifgga2 protein	*Gm4951*	GGCCTGGGAAATAGATCAGG<br>TGTTTCTTCATCTGTAGGTGTGAAC

C-X-C motif chemokine 11	*Cxcl11*	GCGGCTGCTGAGATGAAC<br>CGCCCCTGTTTGAACATAAG
2'-5' oligoadenylate synthetase 1A	*Oas1a*	CAAGCACTGGTACCAACTGTG<br>CAGGCAAAGACAGTGAGCAA
Ubl carboxyl-terminal hydrolase 18	*Usp18*	TTGTTGGGTCACCTGGAAG<br>CCAAGAGATAGGCCGTTTCC
Guanylate-binding protein 3	*Gbp3*	GCCATTGCTGTCTTCATGG<br>TCCTTCCTTTCCTCTATGGTGA
XIAP-associated factor 1	*Xaf1*	GCCATGTGTCTGAGTGCAAA<br>GCAAAGATCACAACGGGTTT
Nuclear autoantigen Sp-100	*Sp100*	GCATATTCCAGAAAACCTCCAA<br>TGATGATAGAGCTCTCAGTGTCAGA
2'-5'-oligoadenylate synthase 1B	*Oas1b*	TGCTGCCAGCCTATGATTTA<br>CCCCATAAAGCAGATCGATAA
Receptor-transporting protein 4	*Rtp4*	GCACCAGCAGACAGTGCTT<br>TGGCACAAGATCATCACCTG
Interferon-gamma-inducible GTPase Ifgga4 protein	*F830016B08Rik*	AGCCTGGAGCACTGTAAAGG<br>AGGAGCTGACCCATGTTGAT
Colony Stimulating Factor 3	*Csf3*	AAGTCCCTGGAGCAAGTGAG<br>CAGCTTGTAGGTGGCACACA
Tenascin C	Tnc	GCTACAAGCTGAAGGTAGAAGGA<br>TGGAGAAGGATCTACCATTGTG
Thrombospondin 1	Thbs1	CACCTCTCCGGGTTACTGAG<br>GCAACAGGAACAGGACACCTA
C-fos-induced growth factor	Figf	GCAACTTTCTATGACACTGAAACAC<br>TCTCTCTAGGGCTGCATTGG
Perilipin 1	Plin1	GATCCCGGCTCTTCAATACC<br>GCAATGGGCACACTGATG
Fatty acid-binding protein 5	Fabp5	ACGGTCTGCACCTTCCAA<br>TGCAGGTGGCATTGTTCA
Acyl-CoA synthetase long chain family member 3	Acsl3	CGGCTGTCTGAAGATCATTG<br>TTCAAAGCTGCCTCTACTTTCC
Acyl-CoA synthetase, bubblegum family, member 1	Acsbg1	GAGCCCAGGTCTGCTTTG<br>CTCCCATGTGGGATGTGG
Angiopoietin-related protein 4	Angptl4	GGGACCTTAACTGTGCCAAG<br>GAATGGCTACAGGTACCAAACC

Krueppel-like factor 5	Klf5	CCGGAGACGATCTGAAACAC<br>CAGATACTTCTCCATTTCACATCTTG
Endothelial PAS domain-containing protein 1	Epas1	CTCCAGGAGCTCAAAAGGTG<br>CAGGTAAGGCTCGAACGATG
Acylglycerol-3-phosphate O-acyltransferase 2, 1-AGP	Agpat2	AAGACGAAGCTCTTCACCTCA<br>TCTGTCAGACCATTGGTAGGG
ATP citrate lyase	Acly	GAAGAAGGAGGGGAAGCTGA<br>TCGCATGTCTGGGTTGTTTA
Acetyl-CoA synthase 2	Acss2	GTGTGATGGCCATACCTTC<br>GTAGTCTGGTGTGGCAATGG

Supplementary Table S2. Sequences of oligonucleptide primers used for ChIP assays

Gene	Region (bp)	Sequence（5′-3′）
Cidec	−1000	GAACTCATTCCCCATGCTCTT
		CCAGGCTTCCCTCCATTTTAT
	−500	ATGACACAAGCTGTACCACAGG
		GCTCACATGCCTGTTTTCCT
	+1	CCTGAGACAGGGACAAATCAGT
		GCCTCACTCACTCCATTGTTT
	+1000	CACCATTGTGGTTCCTGTCAT
		CTTGCCAGTTTCCCCATCTAT
	+2000	ACAGAGAAACCCTGTCTCGAA
		TCACTTGACCATGTCACCTCTC
	+5000	CCTCATCCCACAAATAAACACA
		ACCACCCTATTCCAACAATCC
Gpd1	−1000	AAGGGAGATCAGGCTTATCCA
		AAAGGGAAGGAAGGTCAAAGG
	−500	AGCTACCATCATGGGACATCA
		AAAAGCACTAGCGTCAGCAAC
	+1	TCCCCTTTACTACCCTTTCTG
		TGTGGGCTCCGAGTATTTAAC
	+1000	CCCCCATTAAGCCCCTATATT
		ATGGAGACCCCTCTTTTCCA
	+2000	CAAGCAAGCAAGCAAGCAAG
		CATGCATGCTAGGCAAACTCT
	+5000	ATTGAGCAGCTGGAGAAGGA
		CCTTGTGTTGGAGAATGCTGT

Supplementary Table S3. Pathway analysis on changes on adipocytes treated with TNF-α comparesd with those treated with DMSO

Pathway	Total	Up	Up List	Down	Down List
Osteoclast	3	3	*Dusp1,Saa3,Cxcl5*	0	
Chemokine signaling pathway	3	3	*Ccl2,Ccl7,Cxcl5*	0	
Wnt Signaling Pathway and Pluripotency	1	1	*Fosl1*	0	
EGFR1 Signaling Pathway	1	1	*Dusp1*	0	
Eukaryotic Transcription Initiation	1	1	*Polr2k*	0	
MAPK signaling pathway	1	1	*Dusp1*	0	
Circulating monocytes and cardiac macrophages in diastolic dysfunction	1	1	*Ccl2*	0	
Wnt Signaling Pathway	1	1	*Fosl1*	0	
Adar1 editing defficiency immune response	1	1	*9330175E14Rik*	0	
MAPK signaling pathway	1	1	*Dusp1*	0	
miR-222 in Exercise-Induced Cardiac Growth	1	1	*Mir222*	0	
Spinal Cord Injury	1	1	*Ccl2*	0	
Purine metabolism	1	1	*Polr2l*	0	
miRNA regulation of DNA Damage Response	1	1	*Mir222*	0	
miRNAs involved in DNA damage response	1	1	*Mir222*	0	
Cell Differentiation - Index	1	1	*Mir222*	0	
miRs in Muscle Cell Differentiation	1	1	*Mir222*	0	
Estrogen signaling	1	1	*Polr2k*	0	

Genes with 1.5-fold or over upregulation and those with -1.5-fold or less downregulation in genes with less 0. 2 *p*-value in ANOVA were selected.

Supplementary Table S4. Pathway analysis on changes on TNF-α stimulatus adipocytes co-treated with capric fatty acid (C10) comparesd with those treated with DMSO.

Pathway	Total	Up	Up List	Down	Down List
PodNet: protein-protein interactions in the podocyte	12	3	*Ptgs2,Angptl4,Plaur*	9	*F3,Vdr,Ddr1,Sparc,Cyr61,Sema3f,Tgfb3,Kank2,Cxcl12*
Osteoclast	11	5	*Bcl6,Mmp12,Ereg,Cd36,Scarb1*	6	*F3,Cd109,Fbn1,Mmp2,Efnb2,S1pr3*
Focal Adhesion-PI3K-Akt-mTOR-signaling pathway	10	2	*Csf3,Lipe*	8	*Tnc,Col5a2,Col1a1,Col1a2,Thbs1,Ghr,Figf,Ins2*
PPAR signaling pathway	9	9	*Plin1,Cd36,Fabp4,Fabp5,Acsl3,Acsbg1,Olr1,Angptl4,Scd1*	0	
Focal Adhesion	7	0		7	*Tnc,Figf,Col5a2,Col1a1,Col1a2,Thbs1,Myl6*
Adipogenesis genes	6	5	*Scd1,Fabp4,Klf5,Lipe,*	1	*Ins2*
Insulin Signaling	5	2	*Map3k5,Lipe*	3	*Rhoj,Flot1,Grb14*
Retinol metabolism	5	3	*Scarb1,Cd36,Dhrs3*	2	*Rarg,Aldh1a1*
Myometrial Relaxation and Contraction Pathways	4	1	*Atf3*	3	*Adm,Cnn2,Acta2*
mRNA processing	4	1	*Brca1*	3	*Bicc1,Pcolce,Rbms3*
TGF-beta Receptor Signaling Pathway	4	1	*Atf3*	3	*Tgfb3,Sparc,Vdr*
Endochondral Ossification	4	0		4	*Ghr,Adamts1,Adamts5,Plat*
Metapathway biotransformation	4	2	*Cyp4b1,Ephx2*	2	*Cyp2f2,Gsto2*
Inflammatory Response Pathway	3	0		3	*Col1a1,Col1a2,Thbs1*
Complement and Coagulation Cascades	3	1	*Plaur*	2	*F3,Plat*
Matrix Metalloproteinases	3	1	*Mmp12*	2	*Mmp2,Timp2*
Nuclear receptors in lipid metabolism and toxicity	3	1	*Cyp4b1*	2	*Rarg,Vdr*
Prostaglandin Synthesis and Regulation	3	1	*Ptgs2*	2	*Anxa3,Anxa8*
Fatty Acid Beta Oxidation (streamlined)	3	3	*Acsl3,Cd36,Fabp4*	0	
Fatty Acid Biosynthesis	3	3	*Scd1,Acsl3,Acss2*	0	

MAPK signaling pathway	3	1 *Map3k5*	2	*Tgfb3,Cyp2f2*
Androgen Receptor Signaling Pathway	3	1 *Brca1*	2	*Tgfb1i1,Ghr*
Chemokine signaling pathway	3	0	3	*Ccl6,Cxcl12,Cxcl13*
Hypertrophy Model	3	1 *Atf3*	2	*Cyr61,Ankrd1*
PluriNetWork	3	3 *Klf5,Id1,Brca1*	0	
Non-odorant GPCRs	3	0	3	*Npy1r,S1pr3,Fzd2*
Fatty Acid Beta Oxidation	3	3 *Acss2,Acsl3,Lipe*	0	
Tryptophan metabolism	2	0	2	*Cyp2f2,Aldh1a1*
Amino Acid metabolism	2	1 *Pdk4*	1	*Aldh1a1*
Id Signaling Pathway	2	2 *Id1,Atf3*	0	
Nuclear Receptors	2	0	2	*Rarg,Vdr*
MAPK signaling pathway	2	1 *Map3k5*	1	*Tgfb3*
Cholesterol metabolism (includes both Bloch and Kandutsch-Russell pathways)	2	2 *Acsl3,Scd1*	0	
Oxidative Stress	2	1 *Hmox1*	1	*Sod3*
Triacylglyceride Synthesis	2	2 *Agpat2,Lipe*	0	
Dysregulated miRNA Targeting in Insulin/PI3K-AKT Signaling	2	0	2	*Col1a1,Col1a2*
IL-3 Signaling Pathway	2	1 *Id1*	1	*Mmp2*
Glycolysis	2	1 *Pdk4*	1	*Pdp1*
Eicosanoid Synthesis	2	2 *Ptgs2,Ltc4s*	0	
p53 signaling	2	1 *Rrm2*	1	*Thbs1*
Spinal Cord Injury	2	1 *Mmp12*	1	*Efnb2*
Purine metabolism	2	2 *Rrm2,Pde1b*	0	
Striated Muscle Contraction	2	0	2	*Tpm1,Acta2*
Neural Crest Differentiation	2	1 *Id1*	1	*Pmp22*
GPCRs, Class A Rhodopsin-like	2	1 *Olfr887*	1	*Npy1r*
Oxidation by Cytochrome P450	2	1 *Cyp4b1*	1	*Cyp2f2*
Nucleotide Metabolism	1	1 *Rrm2*	0	
Wnt Signaling Pathway and Pluripotency	1	0	1	*Fzd2*
EGFR1 Signaling Pathway	1	0	1	*Grb14*

Exercise-induced Circadian Regulation	1	1	*Btg1*	0	
Wnt Signaling Pathway NetPath	1	0		1	*Fzd2*
Regulation of Actin Cytoskeleton	1	0		1	*Ins2*
Sphingolipid Metabolism (integrated pathway)	1	0		1	*Ugcg*
Blood Clotting Cascade	1	0		1	*Plat*
Omega-9 FA synthesis	1	1	*Acsl3*	0	
Omega-3/Omega-6 FA synthesis	1	1	*Acsl3*	0	
Eicosanoid metabolism via Cytochrome P450 Mono-Oxygenases (CYP) pathway	1	1	*Ephx2*	0	
Eicosanoid metabolism via Lipo Oxygenases (LOX)	1	1	*Ltc4s*	0	
Eicosanoid metabolism via Cyclo Oxygenases (COX)	1	1	*Ptgs2*	0	
Sphingolipid Metabolism (general overview)	1	0		1	*Ugcg*
TCA Cycle	1	1	*Pdk4*	0	
Eicosanoid Lipid Synthesis Map	1	1	*Ptgs2*	0	
Urea cycle and metabolism of amino groups	1	1	*Odc1*	0	
G1 to S cell cycle control	1	0		1	*Cdkn2c*
GPCRs, Other	1	0		1	*Fzd2*
Wnt Signaling Pathway	1	0		1	*Fzd2*
Mitochondrial LC-Fatty Acid Beta-Oxidation	1	1	*Acsl3*	0	
Wnt Signaling in Kidney Disease	1	0		1	*Fzd2*
Hfe effect on hepcidin production	1	1	*Id1*	0	
TYROBP Causal Network	1	0		1	*Loxl3*
Small Ligand GPCRs	1	0		1	*S1pr3*
p38 MAPK Signaling Pathway	1	1	*Map3k5*	0	
Adar1 editing defficiency immune response	1	0		1	*Ly6a*
ESC Pluripotency Pathways	1	0		1	*Fzd2*
Fatty Acid Omega Oxidation	1	0		1	*Aldh1a1*
Electron Transport Chain	1	1	*Ucp2*	0	

Mecp2 and Associated Rett Syndrome	1	0		1	*Csrp1*
Mir302-367 Promoting Cardiomyocyte Proliferation	1	1	*Lats2*	0	
White fat cell differentiation	1	1	*Klf5*	0	
B Cell Receptor Signaling Pathway	1	1	*Bcl6*	0	
Ovarian Infertility Genes	1	0		1	*Vdr*
Primary Focal Segmental Glomerulosclerosis FSGS	1	1	*Plaur*	0	
Peptide GPCRs	1	0		1	*Npy1r*
G Protein Signaling Pathways	1	1	*Pde1b*	0	
Macrophage markers	1	0		1	*F3*
Cytokines and Inflammatory Response	1	1	*Csf3*	0	
miRNA regulation of DNA Damage Response	1	1	*Brca1*	0	
Alzheimers Disease	1	0		1	*Casp12*
One carbon metabolism and related pathways	1	0		1	*Sod3*
MicroRNAs in Cardiomyocyte Hypertrophy	1	0		1	*Fzd2*
DNA Replication	1	1	*Cdt1*	0	
Odorant GPCRs	1	0		1	*Gpr176*
Diurnally Regulated Genes with Circadian Orthologs	1	1	*Btg1*	0	
ErbB signaling pathway	1	1	*Ereg*	0	
Amino acid conjugation of benzoic a	1	1	*Acss2*	0	
Arachidonate Epoxygenase Epoxide Hydrolase	1	1	*Ephx2*	0	
Keap1-Nrf2	1	1	*Hmox1*	0	
Estrogen signaling	1	1	*Brca1*	0	
TGF Beta Signaling Pathway	1	0		1	*Thbs1*
Statin Pathway	1	1	*Scarb1*	0	

Genes with 1.5-fold or over upregulation and those with -1.5-fold or less downregulation in genes with less 0. 2 p-value in ANOVA were selected.

Supplementary Table S5. Pathway analysis on changes on TNF-α stimulatus adipocytes co-treated with butyric fatty acid (C4) comparesd with those treated with DMSO.

Pathway	Total	Up	Up List	Down	Down List
Adar1 editing defficiency immune response	26	1	*Nfkbia*	25	*Irf7,Ddx58,Tlr3,Dhx58,Oas2,Mx2,Isg15,Tgtp2,Ifi44,Ifit1,Oas1g,Zbp1,Mx1,Oasl2,Gm4951,Cxcl11,Oas1a,Usp18,Ddx60,Gbp3,Xaf1,Sp100,Oas1b,Rtp4,F830016B08Rik*
Osteoclast	10	6	*Il1a,Ccl3,Mmp13,Il6,Ereg,Cxcl5*	4	*Pik3r3,Ctsk,Lrrk1,Vegfb*
mRNA processing	9	0		9	*Sf3a2,Nhp2l1,Trim21,Zbp1,Oas1a,Oas1b,Oas1g,Oas2,Oasl2*
Chemokine signaling pathway	8	6	*Cxcl1,Ccl3,Cxcl2,Cxcl5,Gnb5,Nfkbia*	2	*Cxcl11,Pik3r3*
Toll-like receptor signaling pathway	7	3	*Spp1,Il6,Nfkbia*	4	*Irf7,Cxcl11,Pik3r3,Tlr3*
Spinal Cord Injury	6	6	*Cd47,Il6,Cxcl1,Il1a,Cxcl2,Rhob*	0	
Lung fibrosis	5	5	*Spp1,Il6,Grem1,Ptx3,Ccl3*	0	
TNF-alpha NF-kB Signaling Pathway	5	2	*Nfkbia,Nfkbiz*	3	*Tradd,Usp11,Gab1*
Adipogenesis genes	4	4	*Scd1,Fabp4,Il6,Epa*[illegible]	0	
Focal Adhesion-PI3K-Akt-mTOR-signaling pathway	4	3	*Spp1,Il2ra,Ngf*	1	*Vegfb*
PPAR signaling pathway	4	3	*Fabp4,Olr1,Scd1*	1	*Acox3*
Cytokines and Inflammatory Response	4	4	*Cxcl1,Il1a,Il6,Csf2*	0	
Focal Adhesion	3	2	*Spp1,Rhob*	1	*Vegfb*
IL-2 Signaling Pathway	3	2	*Il2ra,Cd53*	1	*Nmi*
MAPK signaling pathway	3	3	*Ngf,Il1a,Gsta3*	0	
B Cell Receptor Signaling Pathway	3	1	*Nfkbia*	2	*Cmtm3,Gab1*
Androgen Receptor Signaling Pathway	3	1	*Il6*	2	*Tgfb1i1,Prmt1*
PodNet: protein-protein interactions in the podocyte	3	3	*Spp1,Epas1,Nid2*	0	

Apoptosis	3	1	*Nfkbia*	2	*Tradd,Irf7*
Insulin Signaling	2	0		2	*Pik3r3,Gab1*
EGFR1 Signaling Pathway	2	0		2	*Pik3r3,Gab1*
MAPK signaling pathway	2	2	*Ngf,Il1a*	0	
Matrix Metalloproteinases	2	1	*Mmp13*	1	*Mmp11*
Eicosanoid metabolism via Lipo Oxygenases (LOX)	2	1	*Ltc4s*	1	*Acox3*
Cholesterol metabolism (includes both Bloch and Kandutsch-Russell pathways)	2	1	*Scd1*	1	*Ebp*
G1 to S cell cycle control	2	0		2	*Mcm6,Cdkn2c*
GPCRs, Other	2	1	*Olfr394*	1	*Fzd2*
IL-6 signaling Pathway	2	1	*Il6*	1	*Gab1*
Triacylglyceride Synthesis	2	2	*Gpd1,Dgat2*	0	
Myometrial Relaxation and Contraction Pathways	2	2	*Il6,Gnb5*	0	
Prostaglandin Synthesis and Regulation	2	1	*Hsd11b1*	1	*Anxa8*
TYROBP Causal Network	2	1	*Spp1*	1	*Tmem106a*
ESC Pluripotency Pathways	2	0		2	*Gab1,Fzd2*
Electron Transport Chain	2	1	*ND6*	1	*Cox6a2*
Macrophage markers	2	2	*Cd68,Lyz2*	0	
Purine metabolism	2	1	*Ampd3*	1	*Gmpr2*
Heart Development	2	0		2	*Vegfb,Bhlhe40*
Hypertrophy Model	2	2	*Il1a,Ankrd1*	0	
Cell cycle	2	0		2	*Mcm6,Plk1*
PluriNetWork	2	1	*Spp1*	1	*Gab1*
MicroRNAs in Cardiomyocyte Hypertrophy	2	0		2	*Pik3r3,Fzd2*
Endochondral Ossification	2	2	*Spp1,Mmp13*	0	
ErbB signaling pathway	2	1	*Ereg*	1	*Gab1*
Type II interferon signaling (IFNG)	2	0		2	*Tap1,Isg15*
Metapathway biotransformation	2	2	*Fmo2,Fmo3*	0	
Toll Like Receptor signaling	1	0		1	*Tlr3*
Tryptophan metabolism	1	0		1	*Prmt1*

Wnt Signaling Pathway and Pluripotency	1	0		1	*Fzd2*
FAS pathway and Stress induction of HSP regulation	1	1	*Il1a*	0	
Calcium Regulation in the Cardiac Cell	1	1	*Gnb5*	0	
Wnt Signaling Pathway NetPath	1	0		1	*Fzd2*
Regulation of Actin Cytoskeleton	1	0		1	*Pik3r3*
Proteasome Degradation	1	0		1	*Psmb10*
Id Signaling Pathway	1	1	*Ngf*	0	
Glucocorticoid & Mineralcorticoid Metabolism	1	1	*Hsd11b1*	0	
Alpha6-Beta4 Integrin Signaling Pathway	1	0		1	*Pik3r3*
T Cell Receptor Signaling Pathway	1	1	*Fyb*	0	
Inflammatory Response Pathway	1	1	*Il2ra*	0	
Circulating monocytes and cardiac macrophages in diastolic dysfunction	1	1	*Spp1*	0	
Omega-3/Omega-6 FA synthesis	1	0		1	*Acox3*
Eicosanoid metabolism via Cyclo Oxygenases (COX)	1	0		1	*Acox3*
Glycerolipids and Glycerophospholipids	1	1	*Dgat2*	0	
Oxidative Stress	1	1	*Sod2*	0	
Wnt Signaling Pathway	1	0		1	*Fzd2*
Wnt Signaling in Kidney Disease	1	0		1	*Fzd2*
Dysregulated miRNA Targeting in Insulin/PI3K-AKT Signaling	1	0		1	*Pik3r3*
IL-3 Signaling Pathway	1	0		1	*Gab1*
IL-1 Signaling Pathway	1	1	*Il1a*	0	
Parkinsons Disease Pathway	1	0		1	*Uba7*
Microglia Pathogen Phagocytosis Pathway	1	0		1	*Pik3r3*
Fatty Acid Beta Oxidation (streamlined)	1	1	*Fabp4*	0	
Glycolysis	1	1	*Eno3*	0	

p38 MAPK Signaling Pathway	1	0		1 *Tradd*
Fatty Acid Biosynthesis	1	1	*Scd1*	0
Eicosanoid Synthesis	1	1	*Ltc4s*	0
Notch Signaling Pathway	1	0		1 *Dtx3l*
Delta-Notch Signaling Pathway	1	1	*Nfkbia*	0
Limb and Fin Development	1	1	*Grem1*	0
Limb Development	1	1	*Grem1*	0
TGF-beta Receptor Signaling Pathway	1	1	*Stambpl1*	0
G Protein Signaling Pathways	1	1	*Gnb5*	0
miRNA regulation of DNA Damage Response	1	0		1 *Nbn*
Neural Crest Differentiation	1	1	*Rhob*	0
Signaling of Hepatocyte Growth Factor Receptor	1	0		1 *Gab1*
One carbon metabolism and related pathways	1	1	*Sod2*	0
Cytoplasmic Ribosomal Proteins	1	0		1 *Rpl21*
Glycolysis and Gluconeogenesis	1	1	*Eno3*	0
IL-5 Signaling Pathway	1	1	*Nfkbia*	0
DNA Replication	1	0		1 *Mcm6*
Non-odorant GPCRs	1	0		1 *Fzd2*
Folic Acid Network	1	0		1 *Sepw1*
SIDS Susceptibility Pathways	1	1	*Il6*	0
Homologous recombination	1	0		1 *Nbn*
Oxidative phosphorylation	1	1	*ND6*	0
Hypothetical Network for Drug Addiction	1	0		1 *Ppa1*
EBV LMP1 signaling	1	0		1 *Tradd*
TGF Beta Signaling Pathway	1	1	*Spp1*	0

Genes with 1.5-fold or over upregulation and those with -1.5-fold or less downregulation in genes with less 0. 2 *p*-value in ANOVA were selected.
